# Genome-wide identification, molecular evolution and expression analysis of the non-specific lipid transfer protein (nsLTP) family in *Setaria italica*

**DOI:** 10.1186/s12870-022-03921-1

**Published:** 2022-11-28

**Authors:** Feng Li, Kai Fan, Xuhu Guo, Jianxia Liu, Kun Zhang, Ping Lu

**Affiliations:** 1 College of Agronomy and Life Sciences, Shanxi Datong University, Datong, 037009 China; 2 Research and Development Center of Agricultural Facility Technology, Shanxi Datong University, Datong, 037009 China; 3 Key Laboratory of Ministry of Education for Genetics, Breeding and Multiple Utilization of Crops, College of Crop Science, Fujian Agriculture and Forestry University, Fuzhou, 350002 China; 4 Institute of Crop Sciences, Chinese Academy of Agricultural Sciences, Beijing, China

**Keywords:** Foxtail millet (*Setaria italica*), Non-specific lipid transfer proteins (nsLTPs), Phylogenetic classification, Comparative evolutionary analysis, Gene expression profiles

## Abstract

**Background:**

Foxtail millet (*Setaria italica* L.) is a millet species with high tolerance to stressful environments. Plant non-specific lipid transfer proteins (nsLTPs) are a kind of small, basic proteins involved in many biological processes. So far, the genome of *S. italica* has been fully sequenced, and a comprehensive understanding of the evolution and expression of the *nsLTP* family is still lacking in foxtail millet.

**Results:**

Forty-five *nsLTP* genes were identified in *S. italica* and clustered into 5 subfamilies except three single genes (*SinsLTP38*, *SinsLTP7*, and *SinsLTP44*). The proportion of *SinsLTPs* was different in each subfamily, and members within the same subgroup shared conserved exon–intron structures. Besides, 5 *SinsLTP* duplication events were investigated. Both tandem and segmental duplication contributed to *nsLTP* expansion in *S. italica*, and the duplicated *SinsLTPs* had mainly undergone purifying selection pressure, which suggested that the function of the duplicated *SinsLTPs* might not diverge much. Moreover, we identified the *nsLTP* members in 5 other monocots, and 41, 13, 10, 4, and 1 orthologous gene pairs were identified between *S. italica* and *S. viridis*, *S. bicolor*, *Z. mays*, *O. sativa*, and *B. distachyon*, respectively. The functional divergence within the *nsLTP* orthologous genes might be limited. In addition, the tissue-specific expression patterns of the *SinsLTPs* were investigated, and the expression profiles of the *SinsLTPs* in response to abiotic stress were analyzed, all the 10 selected *SinsLTPs* were responsive to drought, salt, and cold stress. Among the selected *SinsLTPs*, 2 paired duplicated genes shared almost equivalent expression profiles, suggesting that these duplicated genes might retain some essential functions during subsequent evolution.

**Conclusions:**

The present study provided the first systematic analysis for the phylogenetic classification, conserved domain and gene structure, expansion pattern, and expression profile of the *nsLTP* family in *S. italica*. These findings could pave a way for further comparative genomic and evolution analysis of *nsLTP* family in foxtail millet and related monocots, and lay the foundation for the functional analysis of the *nsLTPs* in *S. italica*.

**Supplementary Information:**

The online version contains supplementary material available at 10.1186/s12870-022-03921-1.

## Introduction

Foxtail millet (*Setaria italica* L.) originated in China and is the second largest cultivated millet species in the world [1, 2]. It is a diploid (2n = 2x = 18) with an estimated genome size of approximately 515 Mb [1]. Foxtail millet is predominantly cultivated in arid and semiarid regions of the world as food and fodder, and displays remarkable tolerance to abiotic stress [1, 3, 4]. Along with other features such as short life-cycle, small genome, inbreeding nature, and genetic close-relatedness to several bioenergy grasses, foxtail millet has been considered as a favorable candidate for investigating the stress responsive machinery, evolutionary genomics and the system biology of millets and C_4_ panicoid grasses [1]. Thus, the availability of foxtail millet genome information provided excellent opportunity for researchers to initiate whole-genome annotation and perform comparative genomic study in foxtail millet [5]. Until now, foxtail millet has gained popularity among millet research community and several gene families such as AP2/ERF [1], GRAS [2], NF-Y [3], LecRLKs [4], NAC [6], WD40 [7], MYB [8], PPR [9], HSP [10], CDPK [11], BES/BZR [12], and MADS-Box [13] have been identified and characterized to investigate their role in plant abiotic stress tolerance.

Plant non-specific lipid transfer proteins (nsLTPs) are a kind of small, basic proteins, ranging in size from 6.5–10.5 kDa [14–16]. They are abundantly present in various plants, representing up to 4% of the total soluble protein [15]. Plant nsLTPs are able to transfer phospholipids and fatty acids between membranes in vitro, and structurally characterized by an eight cysteine motif (8CM) backbone with the general form C-Xn-C-Xn-CC-CXC-Xn-C-Xn-C [15, 17]. The cysteine residues of these peptides are linked by four disulfide bonds to stabilize a tertiary structure of a hydrophobic cavity [15, 17]. Almost all nsLTPs are synthesized as precursors with an N-terminal secretory signal peptide, thus secreted to the cell exterior for functioning [15, 16, 18]. Based on the molecular mass and connection types between the bonds, the nsLTPs were initially classified into nsLTP1 (9 kDa, Cys_1_-Cys_6_ and Cys_5_-Cys_8_) and nsLTP2 (7 kDa, Cys_1_-Cys_5_ and Cys_6_-Cys_8_). After that, a new classification according to sequence similarity and intervals of 8CM was proposed [17]. The system categorized the 267 *nsLTPs* from rice, wheat and Arabidopsis into nine types (Type I-IX). Then, *nsLTPs* in other plant species such as *Brassica rapa* [18], sorghum [19], cotton [20], tomato [21], tobacco [22] and Solanaceae plants [23] were also grouped according to Boutrot’s method.

In plants, there is considerable evidence showing that nsLTPs play vital roles in a range of biological processes, including cuticular wax and cutin synthesis, seed maturation, and sexual reproduction [15, 18]. On the other hand, nsLTPs also take part in the regulation of signalling and responses to abiotic/biotic stress, such as drought, high salinity, cold stress, and pathogen defense [15, 16, 18]. Thus, nsLTPs are important for plants to withstand various environmental stresses, which cause huge economic loss in agricultural production globally. In previous studies, only a small portion of nsLTPs from foxtail millet have been characterized [24], a genome-wide overview of the nsLTP family in foxtail millet has yet to be reported. Considering the importance of investigating the molecular networks, biological processes, and gene functions of nsLTP proteins, a systematic molecular evolution and expression analysis of the *nsLTPs* in foxtail millet is urgently required. In this study, putative *nsLTPs* were identified in foxtail millet. We conducted a comprehensive study on the phylogenetics, gene structure, genomic location, expansion pattern, and expression profile to evaluate the molecular evolution and biological function of the *nsLTP* family in foxtail millet.

## Results

### Genomic identification and characterization of *S. italica nsLTP* genes

In this study, a total of 45 *nsLTP* genes were identified in *S. italica* and designated as *SinsLTPs*, from *SinsLTP1* to *SinsLTP45* (Additional file 1). The protein structures of the identified SinsLTPs were highly diverse, and the amino acid numbers of mature peptides varied from 67 (SinsLTP13) to 120 (SinsLTP7), with the predicted molecular weight ranging from 6.9 kDa (SinsLTP13) to 12.3 kDa (SinsLTP7). The isoelectric points ranged from 4.29 (SinsLTP11 and SinsLTP12) to 10.25 (SinsLTP16). Besides, we identified 45, 32, 20, 45, and 30 *nsLTP* genes in *S. viridis*, *S. bicolor*, *Z. mays*, *O. sativa*, and *B. distachyon*, respectively, and denoted them as *SvnsLTPs*, *SbnsLTPs*, *ZmnsLTPs*, *OsnsLTPs*, and *BdnsLTPs*, respectively (Additional file 2).

### Phylogenetic analysis

**Fig. 1:**
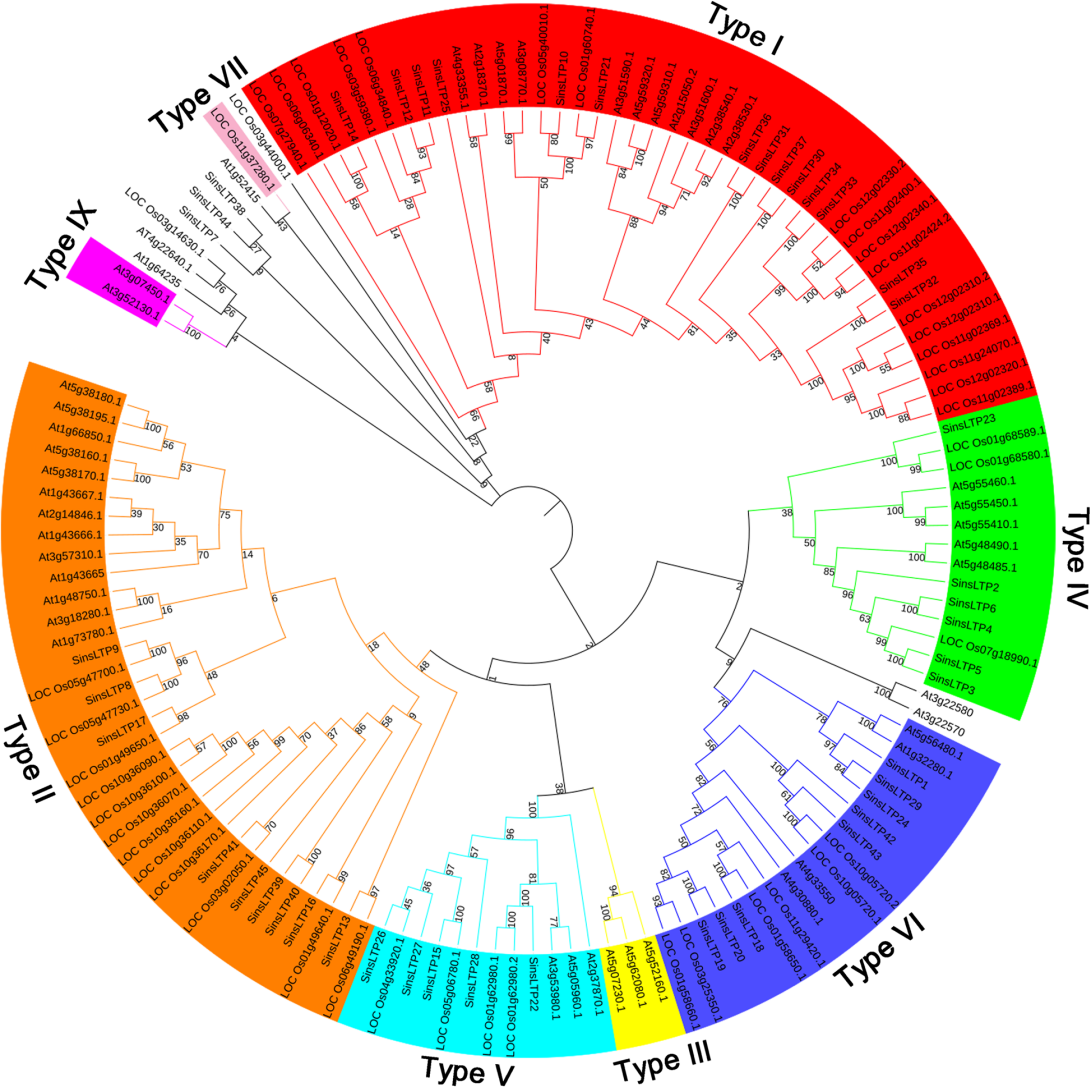
Phylogenetic analysis of the *nsLTP* family from foxtail millet, Arabidopsis, and rice. The neighbor-joining tree was generated using the MEGA7 program based on multiple alignments with ClustalW. The subfamilies are labeled and indicated by different colors, and the numbers in the clades are posterior probability values

**Fig. 2:**
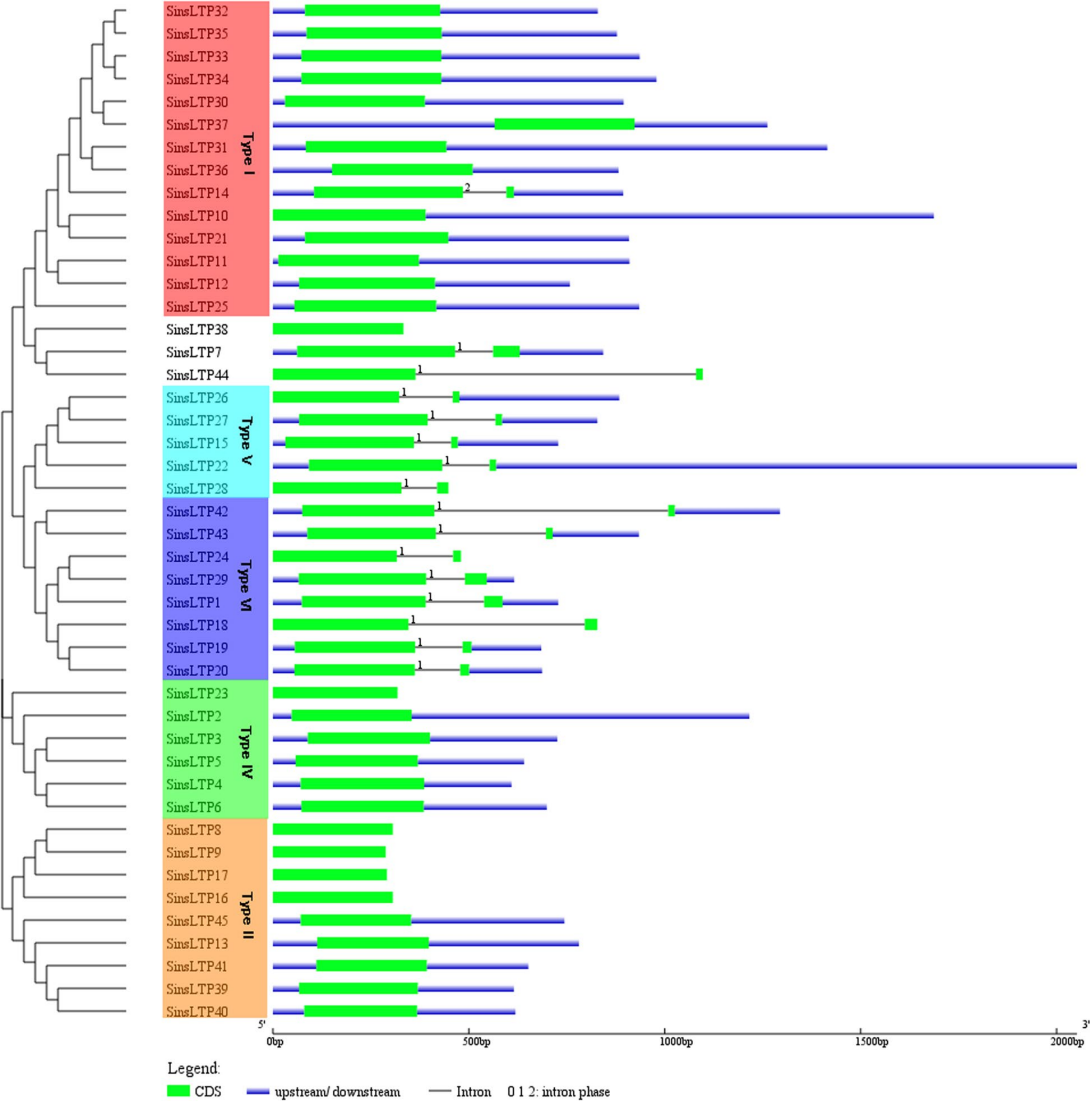
Phylogenetic relationships and gene structures of *nsLTPs* in foxtail millet. Gene structure dynamics of *SinsLTPs* were drawn using the GSDS software. The exons are represented by green boxes and the introns are indicated by black lines. The numbers above the black lines are the intron phase

The identified SinsLTP members were phylogenetically analyzed in this study (Figs. 1 and 2, Additional file 3). According to the previous classification system [17], the SinsLTPs was divided into 5 subfamilies (Type I, Type II, Type IV, Type V, and Type VI), and no Type III, Type VII, and Type IX nsLTPs were identified in *S. italica.* Besides, the member proportion was different in each subfamily. The Type I (31%) had the most genes, followed by Type II (20%), Type VI (18%), and Type IV (13%). Type V (11%) contained the least members (Additional file 4a). In addition, the nsLTPs in *S. viridis*, *S. bicolor*, *Z. mays*, *O. sativa*, and *B. distachyon* were phylogenetically classified (Additional file 5), and a similar member distribution in each subfamily was found in each plant (Additional file 4b-f).

### Exon/intron structures and conserved protein domains of *SinsLTPs*

Gene structure and intron phase were investigated in the *SinsLTP* family (Fig. 2, Additional file 3). Results indicated low diversity in the distribution of intronic regions amid the exonic sequences. Within each subfamily, the intron patterns, formed by relative position and phase, were highly conserved. The number of introns per gene varied from 0 to 1, and no intron was identified in Type II and Type IV *SinsLTPs*.

**Fig. 3:**
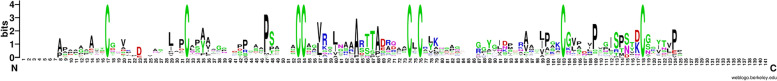
Conserved domain analysis of the SinsLTPs using the WebLogo program. The height of the letter designating the amino acid residue at each position represents the degree of conservation. The numbers on the x-axis represent the sequence positions in the corresponding conserved domains. The y-axis represents the information content measured in bits

The main characteristic of plant nsLTPs is the presence of 8CM in highly conserved positions. In this study, the sequence logos of the identified SinsLTPs were generated to further confirm the conservation of amino acid residues (Fig. 3). It was found that the eight Cys residues were highly conserved in all of the 45 SinsLTPs. Besides, multiple alignments revealed a variable number of inter-cysteine amino acid residues, and 5 nsLTP subfamilies were therefore identified based on the sequence similarity and the typical spacing of the 8CM (Table 1). Between the conserved Cys_1_ and Cys_2_ residues, Type I, II, IV, and VI nsLTPs contained 7–10 residues, while Type V contained 14 residues. Between the conserved Cys_4_ and Cys_5_ residues, Type I nsLTPs contained 19 residues, while the other types contained relatively fewer residues (8–13). Between the conserved Cys_7_ and Cys_8_ residues, Type I nsLTPs contained more residues (13) than Type II nsLTPs (6). In addition, different residues were found in the central position of the Cys_5_XCys_6_ motif. Seven hydrophilic residues (Arg, Gly, Glu, Asn, Ser, Thr, and Lys) and five hydrophobic residues (Leu, Ile, Phe, Val, and Met) existed at the X position of the Cys_5_XCys_6_ motif in the 45 SinsLTPs.

**Table 1:** Diversity of eight cysteine motifs in different subfamilies of nsLTPs identified in *Setaria italica*

Subfamily	Number of members	Spacing pattern												
Type I	14	X_2,3,8_	C	X_9_	C	X_13-15_	CC	X_19_	CXC	X_21-25_	C	X_13_	C	X_4,7_
Type II	9	X_2,3,7–9_	C	X_7_	C	X_12,13_	CC	X_8-10_	CXC	X_23,24_	C	X_6_	C	X_0_
Type IV	6	X_1,5_	C	X_9_	C	X_16,17_	CC	X_9_	CXC	X_21,24,26_	C	X_7,8_	C	X_0,1,3_
Type V	5	X_3,5,9_	C	X_14_	C	X_14_	CC	X_11-13_	CXC	X_24_	C	X_10_	C	X_6,10_
Type VI	8	X_1,3,7,10,16_	C	X_10_	C	X_16-18_	CC	X_9-11_	CXC	X_20,22,23_	C	X_7,9_	C	X_5-8,11,16,19_

Character “X” represents any amino acid, and the Arabic numeral following “X” stands for the numbers of amino acid residues

### Genomic locations and gene duplications of *SinsLTPs*

**Fig. 4:**
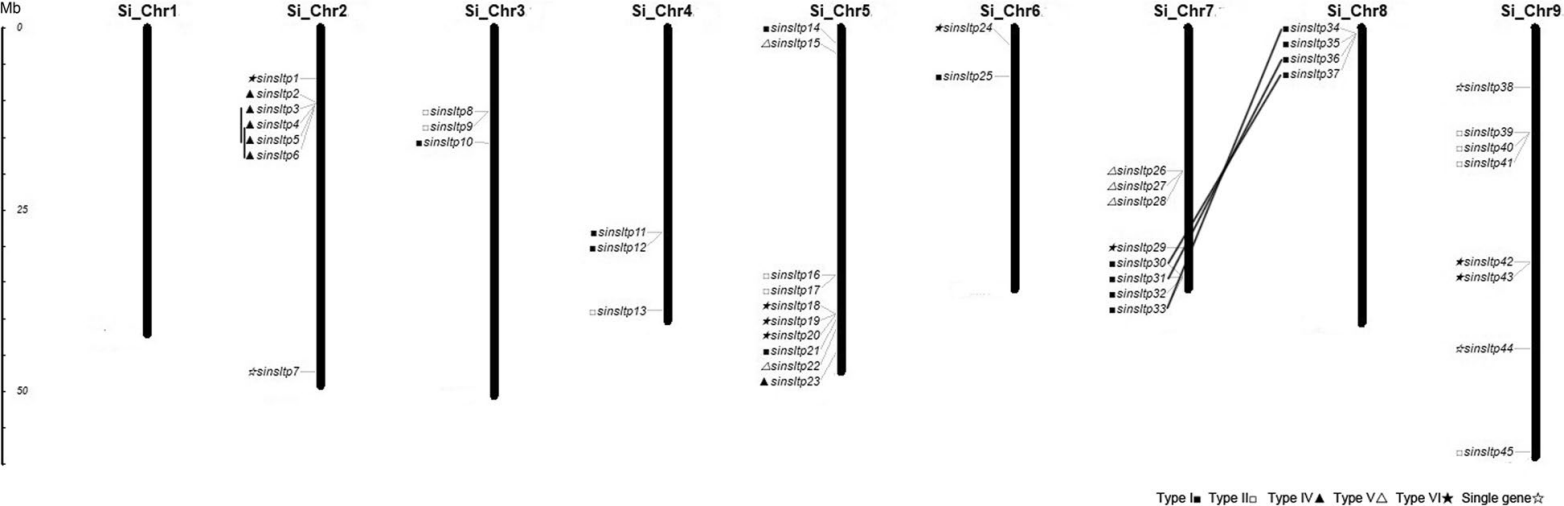
Chromosomal localizations of *nsLTPs* from foxtail millet. The scale represents megabases (Mb). Chromosome numbers are indicated above each vertical bar. The markers before the gene names indicate the *nsLTP* subfamily. The duplicated gene pairs are joined by black lines

To analyze the genomic location of *SinsLTPs* (Additional file 6), the chromosomal distribution diagram of the *SinsLTPs* was generated (Fig. 4). The 45 *nsLTPs* were unevenly distributed on 9 chromosomes, and the number of *nsLTPs* on each chromosome varied widely. Chromosome 5 contained the most *SinsLTPs* with 10 genes, followed by Chromosome 7 and 9 with 8 members. In contrast, only 2 genes were localized on Chromosome 6, and no *nsLTPs* were present in Chromosome 1. Moreover, several *nsLTP* clusters were detected on chromosomes such as the top of Chromosome 8 and the bottom of Chromosome 7 in *S. italica*.

Gene duplication events were investigated to illustrate the expansion of the *SinsLTPs*. In our study, 5 gene duplication events were detected in *S. italica* (Table 2), and the duplication events were concentrated in Type I and Type IV. Meanwhile, based on the sequence analysis and the chromosomal distribution, 2 paired genes were identified to be involved in tandem duplication events, while the other 3 pairs were related to segmental duplication events (Fig. 4, Table 2). In addition, the Ka/Ks ratios of the duplicated *SinsLTPs* were calculated to estimate the molecular evolutionary rates (Table 2). The Ka/Ks ratios of 4 duplicated *SinsLTPs* were less than 1. Moreover, the divergence times between the duplicated gene pairs were analyzed. In *S. italica*, all the Ks values were less than 0.47, and their corresponding duplication age might be less than 36.01 million years ago (MYA).

**Table 2:** Ka/Ks analysis for duplicated gene pairs of *nsLTPs* in *S. italica*

Duplicated gene 1	Duplicated gene 2	Subfamily	Ka	Ks	Ka/Ks	Purifying selection	Duplicate type	Divergence-Time	Age (MYA)
*SinsLTP3*	*SinsLTP5*	Type IV	0.0150	0.4681	0.0321	Yes	tandem	0.0619	36.0085
*SinsLTP4*	*SinsLTP6*	Type IV	0.0413	0.3932	0.1051	Yes	tandem	0.1010	30.2491
*SinsLTP30*	*SinsLTP37*	Type I	0.0034	0.0167	0.2007	Yes	segmental	0.0057	1.2867
*SinsLTP31*	*SinsLTP36*	Type I	0.0207	0.0356	0.5803	Yes	segmental	0.0230	2.7374
*SinsLTP33*	*SinsLTP34*	Type I	0.0233	0.0005	50	No	segmental	0.0198	0.0358

**Fig. 5:**
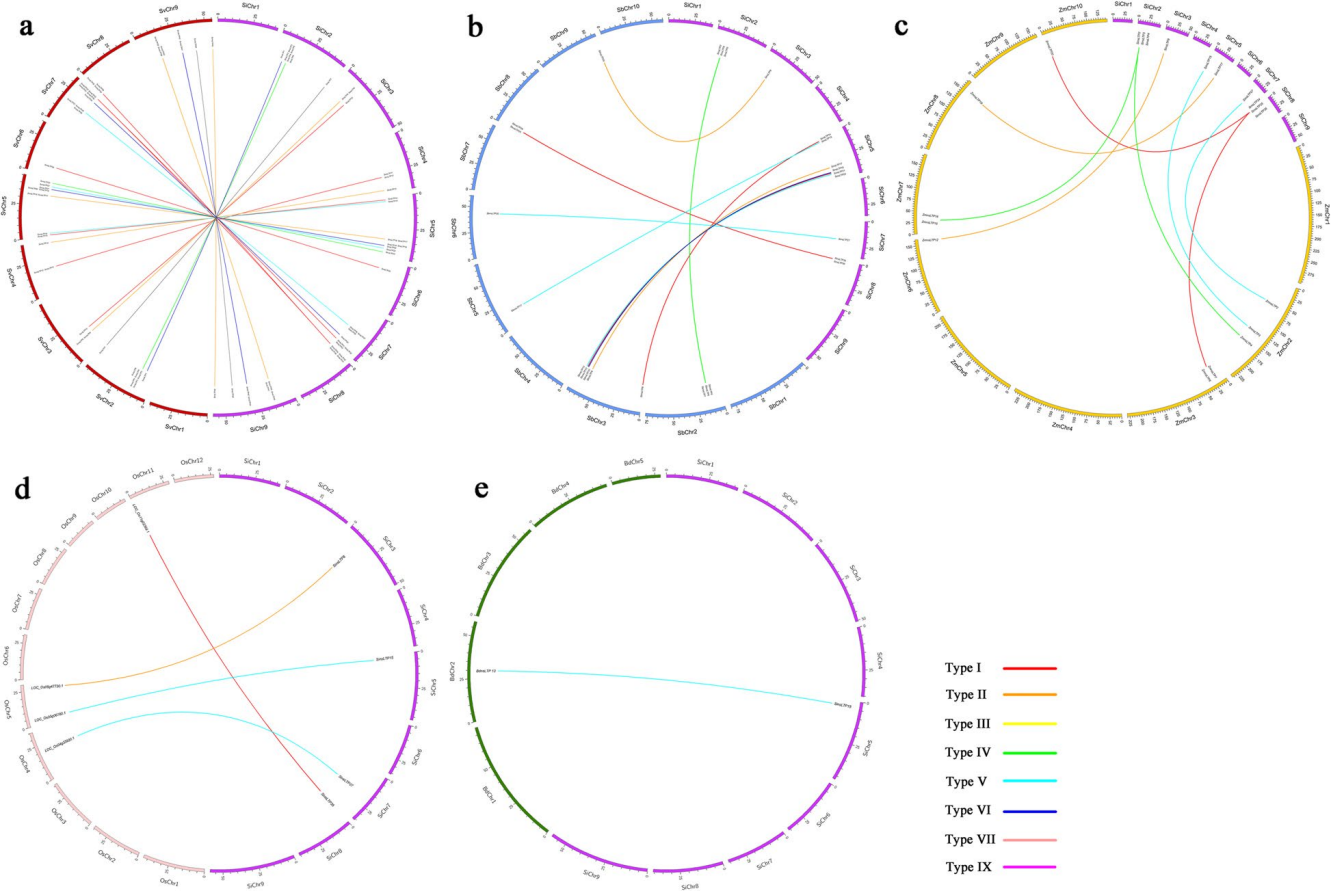
Circos diagram of *nsLTP* orthologous gene pairs between *S. italica* and *S. viridis* (**a**), *S. bicolor* (**b**), *Z. mays* (**c**), *O. sativa* (**d**), and *B. distachyon* (**e**). The orthologous gene pairs are joined by lines. Lines of different colors represent subfamilies within the *nsLTP* family

In our study, orthologous relationships of *nsLTPs* between *S. italica* and 5 other monocots were analyzed, 41, 13, 10, 4, and 1 orthologous gene pairs were identified between *S. italica* and *S. viridis*, *S. bicolor*, *Z. mays*, *O. sativa*, and *B. distachyon*, respectively (Fig. 5, Additional file 7). Of the orthologous gene pairs, most were distributed in Type I, Type II, Type IV, and Type V. All the Ka/Ks ratios except that of 11 orthologous gene pairs between *S. italica* and *S. viridis* were less than 1.

### Regulatory element of *SinsLTPs*

**Fig. 6:**
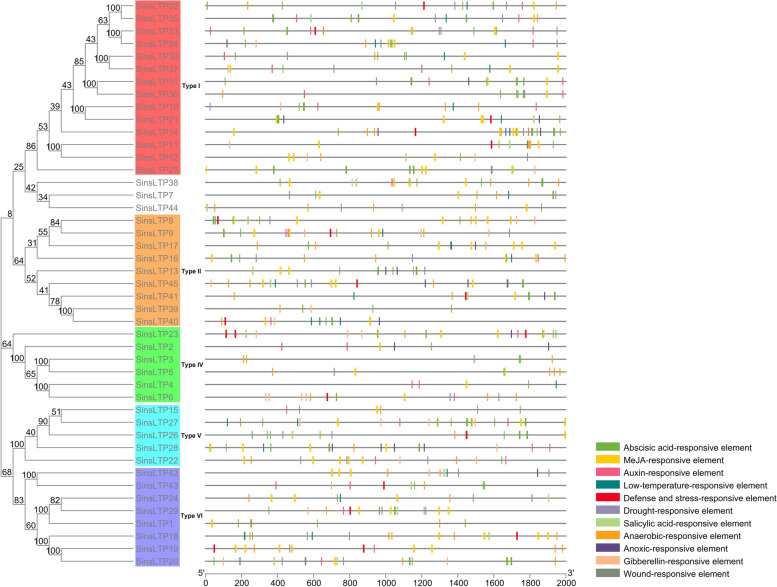
Responsive *cis*-acting elements predicted in the *SinsLTP* promoters. Different colors represent different responsive elements

In this study, *cis*-elements included stress response elements and hormone-related elements were identified in the promoter regions of *SinsLTPs* (Fig. 6, Additional file 8), and the promoter region of *SinsLTPs* from the same subfamily had the similar responsive regulatory elements. Among them, most Type I *SinsLTPs* showed responsive to drought stress, and *SinsLTP33* had the most drought-responsive elements (4), while most Type II members showed responsive cold stress, and *SinsLTP40* contained the most low-temperature-responsive elements (3). Besides, The methyl jasmonate (MeJA)-responsive elements and abscisic acid (ABA)-responsive elements were identified abundantly in the promoter regions of *SinsLTPs* from all subfamilies, and *SinsLTP35* (Type I) and *SinsLTP27* (Type V) contained the most ABA-responsive elements in the promoter region (9), while the most MeJA-responsive elements were 12 in the promoter region of *SinsLTP8* (Type II) and *SinsLTP19* (Type VI). In addition, among the 45 *SinsLTPs*, only two genes, *SinsLTP25* (Type I) and *SinsLTP20* (Type VI) had a wound-responsive element.

### Tissue-specific expression pattern of *SinsLTPs* using RNA-seq data and qRT-PCR

**Fig. 7:**
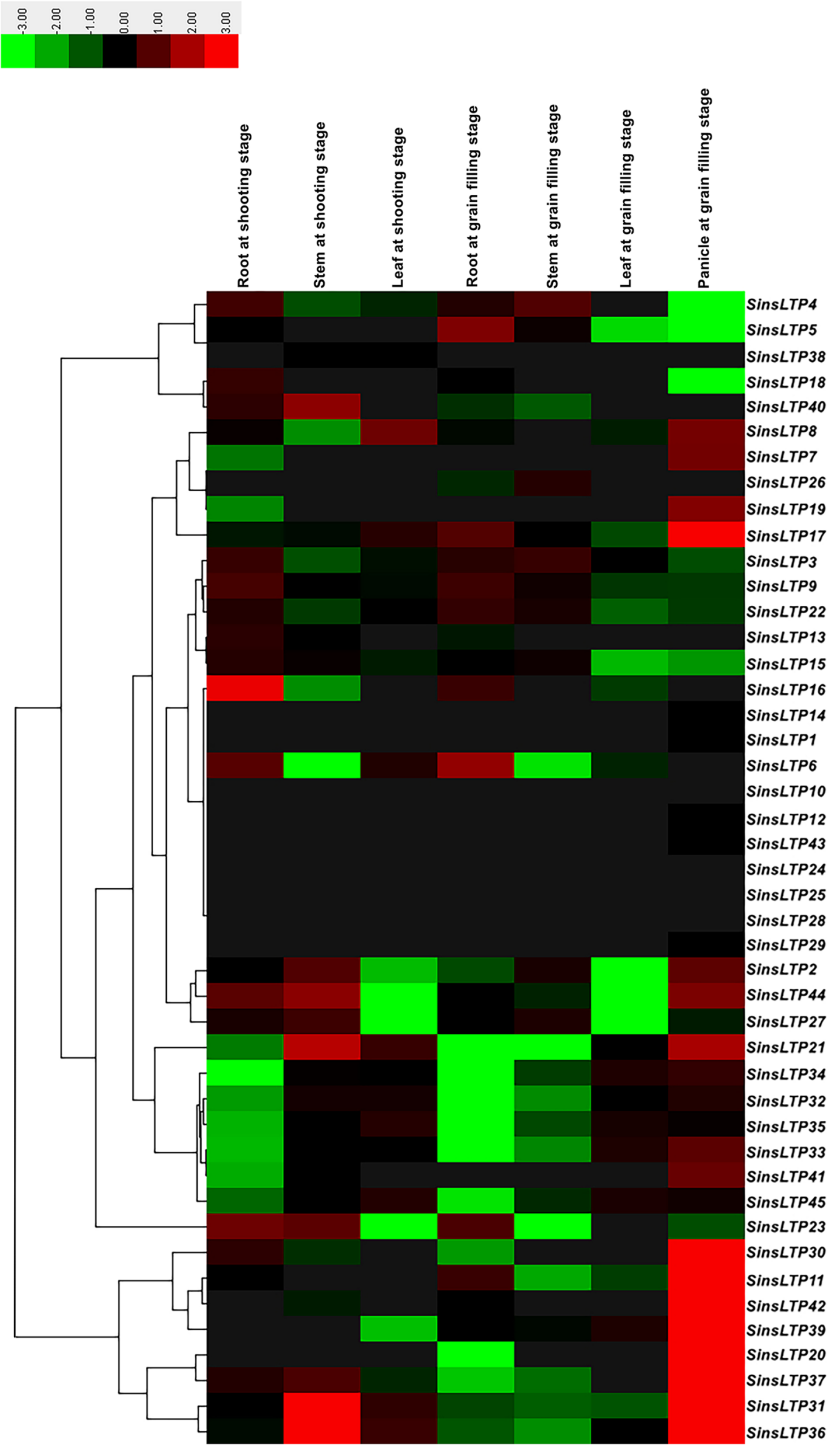
Hierarchical clustering of tissue-specific expression of *SinsLTP* genes from RNAseq data. The colored bars represent the relative signal intensity values

The RNA-seq data in the SRA database at NCBI were used to examine the expression profiles of the *SinsLTPs*, and an expression heatmap in 7 different tissues was mapped (Fig. 7). Generally, most *SinsLTPs* had a broad expression spectrum, and 4 *SinsLTPs* (*SinsLTP10*, *SinsLTP24*, *SinsLTP25*, and *SinsLTP28*) had trace or no detected expression in the 7 tissues. Besides, *SinsLTPs* showed similar tissue-specific expression levels within the same subfamily. *SinsLTPs* in Type IV and Type V subfamily expressed at high levels in roots and stems, while *SinsLTPs* in Type I shared high expression levels in stems and leaves. Most Type VI *SinsLTPs* expressed predominately in flower organs (panicle). In addition, some genes such as *SinsLTP3*, *SinsLTP9*, *SinsLTP15*, and *SinsLTP45* showed high transcript level in all tissues while the expression level of other genes such as *SinsLTP8*, *SinsLTP13*, *SinsLTP16*, and *SinsLTP26* was extremely low.

**Fig. 8:**
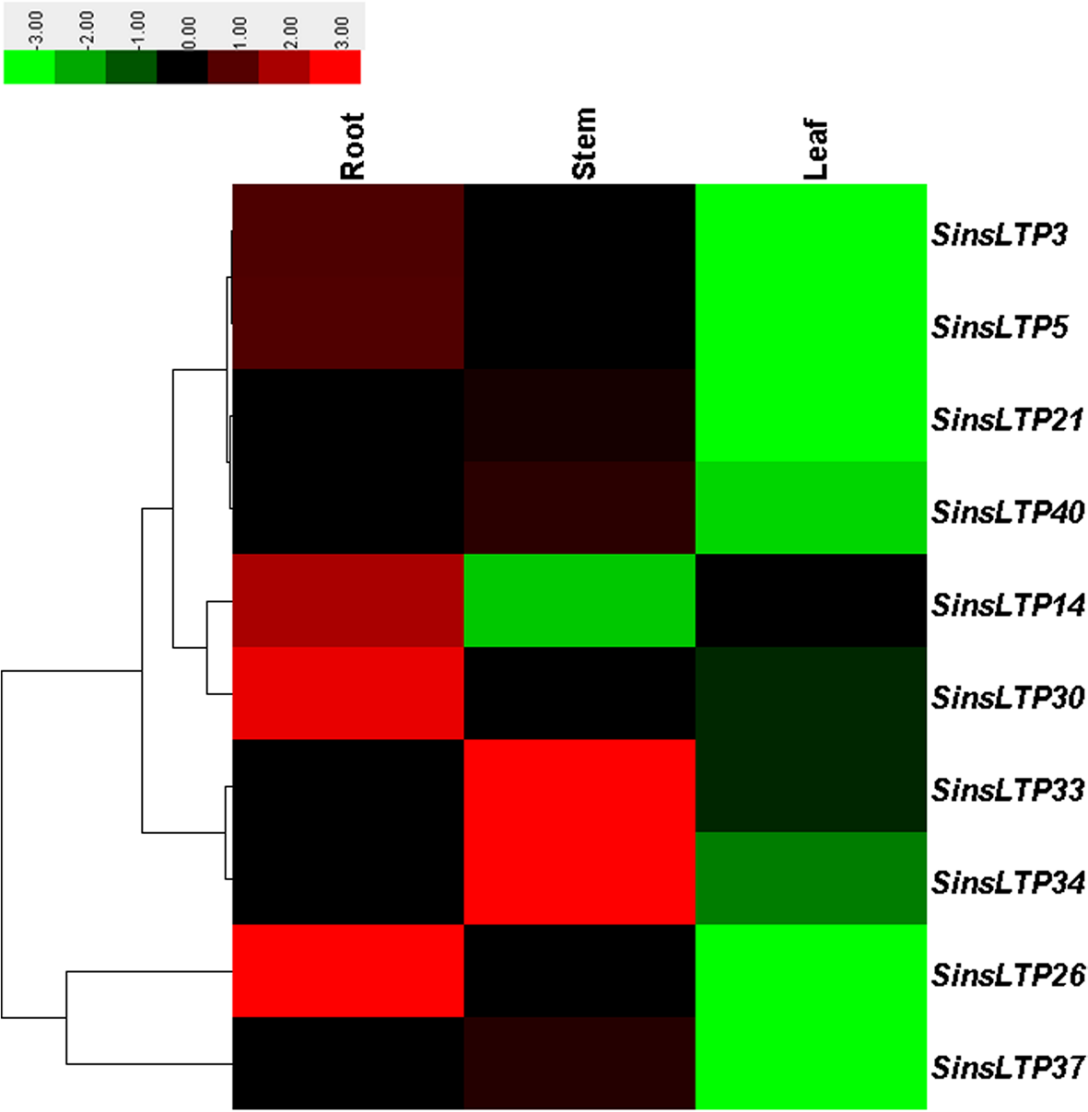
Expression profile of 10 *SinsLTP* genes in roots, stems, and lesves using qRT-PCR. The colored bars represent the relative signal intensity values

Using qRT-PCR, we analyzed the expression of 10 selected *SinsLTP* genes in roots, stems and leaves of Yugu No.1 seedlings. As shown in Fig. 8, *SinsLTP3*, *SinsLTP5*, and *SinsLTP30* were expressed at relatively high levels in the root, while *SinsLTP21*, *SinsLTP33*, *SinsLTP34*, *SinsLTP37*, and *SinsLTP40* showed high transcript level in the stem. The qRT-PCR results are consistent with the former RNA-seq data available in the public database (Fig. 7).

### Expression profiling of *SinsLTPs* during abiotic stresses treatments

**Fig. 9:**
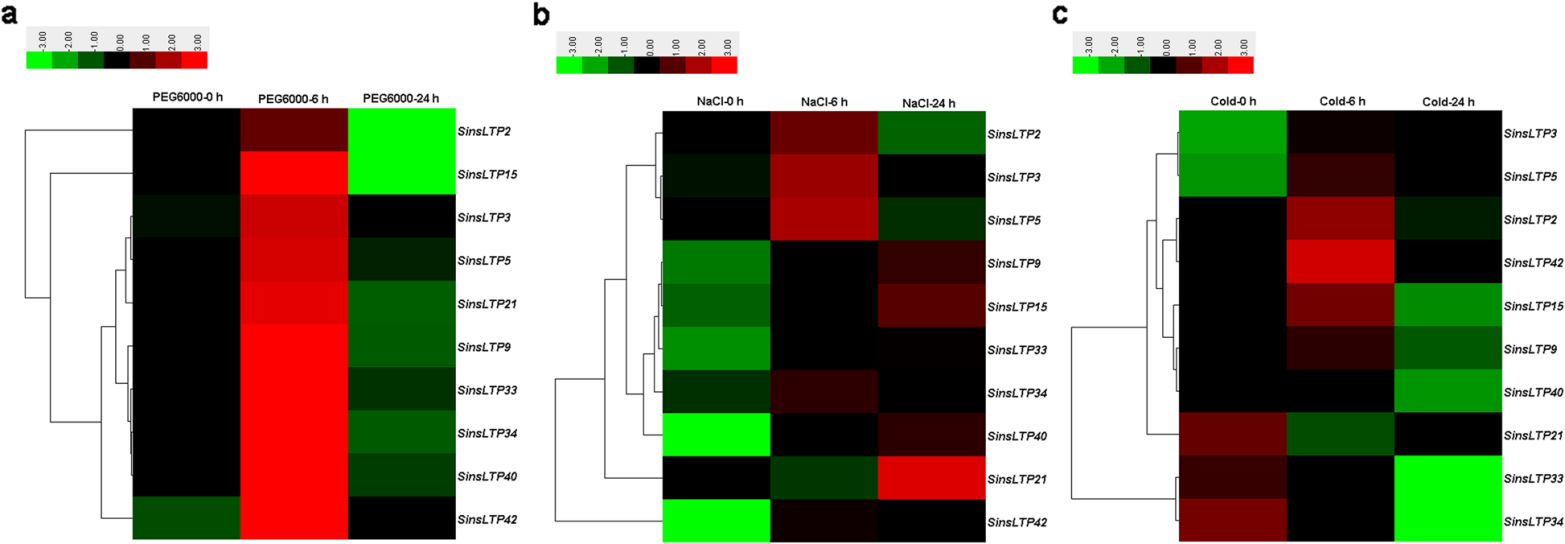
Heat map representation and hierarchical clustering of *SinsLTPs* under drought (**a**), salt (**b**), and cold (**c**) stress from qRT-PCR experiment. The color bar represents the relative signal intensity value

In the current study, a total of 10 *SinsLTPs* from all the subfamilies were selected to investigate the expression patterns of *SinsLTPs*. The result showed that the expressions of all the 10 *SinsLTPs* were induced after drought and salt stress, while some *SinsLTPs* such as *SinsLTP21*, *SinsLTP33*, *SinsLTP34*, and *SinsLTP40* were repressed after cold stress (Fig. 9). Moreover, the expression patterns of 2 duplicated *SinsLTP* gene pairs (*SinsLTP3*/*SinsLTP5* and *SinsLTP33*/*SinsLTP34*) were compared (Additional file 9), and the 2 paired genes shared almost equivalent expression profiles after drought, salt and cold stress treatment.

## Discussion

As a model monocot, the announcement of foxtail millet genome sequencing offers a good opportunity to further investigate the monocot and plant evolution in general. The present study mainly analyzed the molecular evolution and expression pattern of *SinsLTPs*. In this study, a total of 45 *nsLTP* gene members were identified in *S. italica*, and 45, 32, 20, 45, and 30 *nsLTP* genes in *S. viridis*, *S. bicolor*, *Z. mays*, *O. sativa*, and *B. distachyon*, respectively. The encoded proteins of SinsLTPs showed significant differences in physical and chemical properties (Additional file 1), which were comparable with nsLTPs from other plant species [17–23]. The phylogenetic classification of nsLTPs provided comprehensive information about the gene family and facilitated further functional analysis. In the current study, the identified *nsLTPs* in the 6 monocots were divided into 7 subfamilies (Additional file 5), and a similar member distribution in each subfamily was found in each plant (Additional file 4). However, not all the subgroups were present in each plant. No Type III and VII *nsLTPs* were found in *S. italica*, *S. viridis*, and *B. distachyon*, and no Type VII *nsLTPs* existed in *S. bicolor*. In addition, previous studies have reported that Type VII *nsLTPs* were unique to monocots while Type IX *nsLTPs* appeared specifically in dicots [17, 20, 23]. In our study, the *nsLTPs* in *Z. mays* and *O. sativa* further confirmed this viewpoint, while *S. italica*, *S. viridis*, *S. bicolor*, and *B. distachyon* lost Type VII members. The result suggested that the evolution of plants not only involves gene retentions, but also is accompanied by gene losses and mutations [20]. Moreover, the proportion of *nsLTPs* in each subfamily indicated that Type I seemed to have contracted while Type II expanded in *S. bicolor* and *Z. mays* compared with 4 other monocots (Additional file 4). The gene retentions and losses might be associated with the related functions during plant evolution [20, 25].

The intron–exon pattern carries the imprint of the evolution of a gene family [26, 27]. In this study, the gene structures of *SinsLTPs* were highly conserved within each subgroup. Besides, the number of introns of *SinsLTPs* varied from 0 to 1, and no intron was found in Type II and Type IV genes (Fig. 2). Our result showed some differences from other studies, which demonstrated the generality that some *nsLTPs* in Type IV contained introns [17, 18, 28]. As intron loss events have been considered the main cause for the formation of new *nsLTP* types, and contributed to the formation of novel genes within the specific gene subgroups [28], Type IV *nsLTP* genes in *S. italica* might have evolved with no introns contained compared with the *nsLTPs* in other plants. Previous studies have indicated that the *nsLTP* family evolved in early diverged land plants, and during land plant evolution, novel types of *nsLTPs* generated to help plants adapt to environmental changes on land gradually [15, 28].

Like other plant nsLTPs, the nsLTPs identified in *S. italica* showed the presence of 8CM in highly conserved positions (Fig. 3, Table 1). In previous studies, the properties of the amino acid may determine the Cys pairing style, thus influencing the overall folding of nsLTPs [18, 28]. Generally, a hydrophilic amino acid existed separating the Cys_5_ and Cys_6_ of nsLTP1, whereas a hydrophobic residue was present in the Cys_5_XCys_6_ motif of nsLTP2 [18, 28]. In our study, the Cys_5_XCys_6_ of Type I nsLTPs harbored a hydrophilic residue in the central position, while the other subfamilies contained a hydrophobic residue in the same position. Among the five hydrophobic residues existed in the Cys_5_XCys_6_ motif, Leu was present most frequently (64.52%), and this result is consistent with previous studies [17, 18, 20].

It has been recognized that gene duplication plays a critical role in the genesis of evolutionary novelty and complexity [29, 30]. To elucidate the expanded mechanism of the *nsLTP* gene family in *S. italica*, gene duplication events were investigated in this study (Fig. 4, Table 2). We identified 5 duplicated *SinsLTP* gene pairs, and the duplication events were unevenly distributed across the *SinsLTP* subfamilies. The preferential lineage-specific expansion of Type I and Type IV subfamilies in *S. italica* may be associated with the expansion of the *nsLTP* family. Besides, the Ka/Ks ratios for 4 duplicated *SinsLTPs* were less than 1 (Table 2), indicating that the *SinsLTP* members mainly experienced purifying selection with limited functional divergence [20, 25], which was supported by their expression profiles (Additional file 9). The 2 paired genes (*SinsLTP3*/*SinsLTP5* and *SinsLTP33*/*SinsLTP34*) shared similar expression patterns in response to drought, salt, and cold stress. These results indicated that these duplicated genes might have retained some essential functions during subsequent evolution; indeed, most duplicated plant genes are known to have similar evolutionary fates [20, 31–33]. It is possible that the regulatory regions, upstream of the gene, have been duplicated along with the coding region of *sinsLTPs*, resulting in a similar expression pattern between the duplicated genes [34–36]. Meanwhile, the results in our study indicated that both tandem and segmental duplication events contributed to the expansion of the *nsLTP* family in *S. italica* (Fig. 4, Table 2), and most of the *nsLTP* duplication events in *S. italica* might have occurred fewer than 36.01 MYA.

Of plants with sequenced genomes, *S. italica* and *S. viridis* are the closest relatives, together with sorghum and maize, they all belong to Panicoideae subfamily, and are suited for studies of C_4_ evolution and comparative grass genomics. In the present study, Sequence comparison of *SinsLTP* genes with other grasses like foxtail millet, sorghum, maize, as well as the two graminaceous model, rice and *B. distachyon* were performed and the distribution of orthologous *nsLTP* genes were displayed (Fig. 5). The result demonstrated that 41, 13, 10, 4, and 1 *SinsLTPs* had orthologs in *S. viridis*, *S. bicolor*, *Z. mays*, *O. sativa*, and *B. distachyon*, respectively (Fig. 5, Additional file 7), taking the evolutionary tree (Additional file 5) constructed into consideration, *S. italica* and *S. viridis nsLTPs* were phylogenetically closely related compared with the *nsLTPs* in other grass crops, which was in accordance with expectations [5]. Besides, all Ka/Ks ratios calculated indicated the functional divergence within the *nsLTP* orthologous genes was limited.

As for multigene families, gene expression analysis often provides useful clues for function prediction. The tissue-specific expression patterns of *SinsLTPs* obtained from RNA-seq data (Fig. 7) and qRT-PCR (Fig. 8) indicated their important roles in performing diverse developmental and physiological functions. Among them, Type V *nsLTPs* expressed primarily in the vascular bundles, and they were deduced to be involved in signal transduction [19, 20]. Besides, Type VI *nsLTPs* showed flower-specific expression pattern in *S. italica*, indicating that Type VI members play an inportant role in flower development [37]. Moreover, foxtail millet has been studied as a model to understand drought, salt, and cold tolerance in plants, and *nsLTP* genes identified in various plant species have been proven to play crucial roles in abiotic stress response [38–42]. With the goal of identifying candidate abiotic stress-responsive *SinsLTP* genes, the analysis of expression profiles of selected *SinsLTP* genes was performed in the current study. As shown in Fig. 9, all the 10 selected *SinsLTPs* were responsive to drought, salt, and cold stress. Among them, *SinsLTP40* is orthologous to *OsLTPL159* [40], *SinsLTP33* and *SinsLTP34* are orthologous to *LTP3* [41], and *SinsLTP42* is orthologous to *OsDIL* [42], *SinsLTP2*, *SinsLTP3*, and *SinsLTP5* are orthologous to *DIR1* [43]. These genes mentioned above have been reported to play a role in defense signaling. Additionally, in terms of the promoter elements identified in the 10 *SinsLTPs* (Fig. 6), all the genes contained the regulatory elements responsive to stress or hormone, which is in line with their expression pattern to stress treatment (Fig. 9). These genes showed responsive to abiotic stress, and can be selected as candidate genes for further characterization in their functional involvement in plant resistance to abiotic stress.

## Conclusions

In summary, this study identified 45 *nsLTPs* in foxtail millet, and comprehensively analyzed the important features of the gene family such as phylogenetic classification, expansion pattern, and expression profile. The present study deepened our understanding of the molecular evolution and expansion pattern of the *nsLTP* family in foxtail millet, and provided candidate genes for accelerating the genetic improvement of crops.

## Materials and methods

### Genomic identification of non-specific lipid transfer proteins

The genomic sequences of *S. italica*, *S. viridis*, *S. bicolor*, *Z. mays*, *O. sativa*, and *B. distachyon* were downloaded from the Phytozome database (https://phytozome-next.jgi.doe.gov). The Arabidopsis nsLTP amino acid sequences were obtained from the Arabidopsis Information Resource (TAIR) (http://www.arabidopsis.org) and were used as queries by searching against the above-mentioned sequences using the BLASTP program with default parameters [44]. Then, these putative sequences were further verified to contain the conserved LTP domain using the Conserved Domain Database (CDD) program (https://www.ncbi.nlm.nih.gov/cdd). Afterwards, the candidate nsLTP sequences were manually screened step by step as described by previous studies [18, 20], and the rest of the nsLTPs were finally confirmed and used for the following analysis.

### Phylogenetic classification and structural analysis

A multiple sequence alignment of the nsLTP sequences was generated using the ClustalW program [45]. Then, the neighbor joining (NJ) phylogenetic trees were constructed by MEGA7 with 1000 bootstrap iterations [46]. The alternatively spliced forms of *S. italica nsLTPs* were obtained from the Phytozome database, and the genomic schematic diagrams of the *nsLTPs* were visualized using the GSDS tool (http://gsds.cbi.pku.edu.cn/). Sequence logos of the conserved nsLTP subdomains were generated with the WebLogo program (http://weblogo.berkeley.edu/). Primary and secondary protein structures were predicted with ProtParam (http://web.expasy.org/protparam/) and SOPMA (https://npsa-prabi.ibcp.fr/cgi-bin/npsa_automat.pl?page=npsa%20_sopma.html).

### Chromosomal mapping and gene duplications

The chromosomal location information of the *nsLTPs* from *S. italica*, *S. viridis*, *S. bicolor*, *Z. mays*, *O. sativa*, and *B. distachyon* were extracted from the Phytozome database. Duplicated gene pairs were searched via BLASTP and phylogenetic analysis according to the previous report [47]. Briefly, the length of aligned sequence cover was > 80% of the longer gene, and the identity of the aligned regions was > 80%. Besides, only one duplication event was counted for tightly linked genes. The chromosomal distribution images of the *SinsLTPs* were generated using the MapInspect software (http://mapinspect.software.informer.com), and the segmental and tandem duplication events were defined based on the chromosomal locations of the genes. The orthologous *nsLTP* genes between *S. italica* and other monocots were plotted with the Circos program [48].

The evolutionary rates, Ka (non-synonymous substitution rate) and Ks (synonymous substitution rate) were estimated using the KaKs_Calculator package [49], and the Ka/Ks ratio was calculated to assess the selection pressure for each duplicated gene pair. Time (million years ago, MYA) of divergence of duplicated *SinsLTPs* was estimated using the formula “t = Ks/2r”, and a neutral substitution rate (r) of 6.5 × 10^−9^ was used in the current study [6].

#### Promoter analysis

The promoter sequences comprising 2000 bp of the upstream regions of *SinsLTPs* were extracted from the Phytozome database. Potential responsive regulatory elements of the extracted sequences were predicted with the PlantCARE database (http://bioinformatics.psb.ugent.be/webtools/plantcare/html/) [50]. The distribution of the responsive regulatory elements was visualized using the TBtools software (https://github.com/CJChen/TBtools) [51].

### Tissue-specific expression profile of *SinsLTPs* using RNA-seq data

Publicly available RNA-seq data of foxtail millet were downloaded from the NCBI-SRA database (https://www.ncbi.nlm.nih.gov/). Each RNA-seq sample had a clear annotation and its corresponding biological replicates. The RNA-seq data were derived from 7 tissues in *S. italica* (SRX13556037-SRX13556057), and high-quality RNA-seq data were obtained using the Trimmomatic software [52]. After that, the RNA-Seq reads were mapped to the reference *S. italica* genome, and the gene expression data were calculated using the pipeline of HISAT, StringTie, and Ballgown [53]. The expression level was log-transformed via the log_2_^(FPKM+1)^ function by using the values of fragments per kilobase per million read (FPKM) to reduce mean–variance dependency [54]. Lastly, the median of the expression levels of replicated samples was calculated, and the expression levels were clustered using the Cluster 3.0 software [55].

### Plant materials, growth conditions and stress treatments

Seeds of foxtail millet cultivar Yugu No.1 obtained from Institute of Crop Sciences, Chinese Academy of Agricultural Sciences were cultivated in a growth chamber at controlled conditions (28 °C day/ 23 °C night, 14 h light/10 h dark). For tissue specific expression pattern anslysis, roots, stems, and leaves of 21-day-old seedlings were harvested. For stress treatments, 21-day-old seedlings were exposed to 250 mM NaCl (salinity), 20% PEG 6000 (dehydration) and 4 °C temperature (cold) for 6 h (early) and 24 h (late). Unstressed plants were maintained as controls. After the treatments, seedlings were immediately frozen in liquid nitrogen and stored at -80 °C until RNA isolation.

### RNA isolation and quantitative real-time PCR

Total RNA was extracted from the collected samples using the EZNA® Plant RNA Kit (Omega Bio-tek, USA), and cDNA was prepared using HiScript®ll Q RT SuperMix for qPCR(+ gDNA wiper) Kit (Vazyme). For the quantitative real-time PCR, gene-specific primers were designed (Additional file 10) and synthesized commercially (Qinke, Beijing, China). The qRT-PCR analysis was performed in the Roche LightCycler480 Real-Time PCR system by ChamQ Universal SYBR qPCR Master Mix (Vazyme), The cDNAs were amplified over 40 cycles with an annealing temperature of 60 °C. The amounts of transcript accumulated for *SinsLTP* genes normalized to the internal control Actin (AF288226.1) were determined using the 2^−⊿⊿Ct^ method [56]. Each experiment was repeated in triplicate using independent RNA samples. The expression profiles of the *SinsLTPs* were clustered using the Cluster 3.0 software [55].

## Supplementary Information

**Additional file 1. **The structural analysis of nsLTPs identified in *S. italica.*

**Additional file 2. **The *nsLTPs* identified in *S*. *viridis*, *S.* *bicolor*, *Z.* *mays*, *O.* *sativa* and *B.* *distachyon* in this study.

**Additional file 3. **The classification and gene structures of *nsLTPs* in *S. italica.*

**Additional file 4. **The percentage of members in each *nsLTP* subfamily in *S.* *italica* (**a**), *S.* *viridis* (**b**), *S.* *bicolor *(**c**), *Z.* *mays* (**d**), *O.* *sativa* (**e**) and* B.* *distachyon *(**f**).

**Additional file 5. **Phylogenetic relationships of the nsLTPs in *S. italica*,* S. viridis*,* S. bicolor*, *Z. mays*,* O. sativa*,* B. distachyon* and Arabidopsis. Amino acid sequences were aligned using ClustalW and the neighbor-joining tree was generated through the MEGA7 program. The subfamilies are labeled and denoted by different colors and the numbers at nodes represent bootstrap support values from 1000 replicates.

**Additional file 6. **Genomic locations of *nsLTPs *in* S. italica*.

**Additional file 7. **Ka/Ks analysis for orthologous *nsLTP* gene pairs between between *S. italica* and *S. viridis*,* S. bicolor*, *Z. mays*,* O. sativa *and* B. distachyon.*

**Additional file 8. **Number of the responsive-regulatory elements in the promoter regions of *SinsLTPs.*

**Additional file 9. **Expression patterns of some duplicated *SinsLTP* genes after drought (**a, b**), salt (**c, d**) and cold stress treatment (**e, f**).

**Additional file 10. **PCR primers used for qRT-PCR in this study.

## Data Availability

The Arabidopsis nsLTP amino acid sequences were obtained from the Arabidopsis information source (TAIR) database (http://www.arabidopsis.org). The genomic sequences of *Setaria italica*, *Setaria viridis*, *Sorghum bicolor*, *Zea mays*, *Oryza sativa*, and *Brachypodium distachyon* were downloaded from the Phytozome database (https://phytozome-next.jgi.doe.gov). The RNA-seq data of *S. italica* (SRX13556037-SRX13556057) were downloaded from the NCBI-SRA database (https://www.ncbi.nlm.nih.gov/). All data used during the current study are included in this published article and its additional files or are available from the corresponding author on reasonable request.

## Abbreviations

nsLTP: Non-specific Lipid Transfer Protein;
8CM: Eight Cysteine Motif;
NCBI: National Center for Biotechnology Information;
TAIR: The Arabidopsis Information Resource;
CDD: Conserved Domain Database;
MEGA: Molecular Evolutionary Genetics Analysis;
GSDS: Gene Structure Display Server;
Ka/Ks: Non-synonymous Substitution Rate/Synonymous Substitution Rate;
SRA: Sequence Read Archive;
FPKM: Fragments per Kilobase per Million Read;
qRT-PCR: Quantitative Real-time PCR
