# Supplementary material for: Genome-wide identification, molecular evolution and expression analysis of the non-specific lipid transfer protein (nsLTP) family in Setaria italica
Source: BMC Plant Biol. 2022 Nov 28;22:547. doi: 10.1186/s12870-022-03921-1 (PMC9703814; doi:10.1186/s12870-022-03921-1)
Supplement: Supplementary file 1 — Additional file 1. The structural analysis of nsLTPs identified in S. italica. [file 12870_2022_3921_MOESM1_ESM.docx]

**Additional file 1:** The structural analysis of nsLTPs identified in *S. italica*

| Name | Locus name | Protein length (AA) | Signal peptide length (AA) | Mature peptide length (AA) | Alpha helix | Extended strand | Beta turn | Random coil | Molecular weight | Theoretical pI |
| --- | --- | --- | --- | --- | --- | --- | --- | --- | --- | --- |
| SinsLTP1 | Seita.2G079800 | 120 | 26 | 94 | 50 | 13 | 4 | 53 | 10785.33 | 5.74 |
| SinsLTP2 | Seita.2G106500 | 101 | 24 | 77 | 39 | 10 | 5 | 47 | 7868.05 | 4.78 |
| SinsLTP3 | Seita.2G106800 | 103 | 26 | 77 | 40 | 9 | 2 | 52 | 8116.49 | 8.74 |
| SinsLTP4 | Seita.2G106900 | 104 | 26 | 78 | 52 | 7 | 2 | 43 | 8263.61 | 6.71 |
| SinsLTP5 | Seita.2G107000 | 103 | 26 | 77 | 50 | 5 | 5 | 43 | 8109.50 | 8.74 |
| SinsLTP6 | Seita.2G107100 | 103 | 25 | 78 | 39 | 11 | 7 | 46 | 8179.53 | 6.71 |
| SinsLTP7 | Seita.2G417500 | 157 | 37 | 120 | 37 | 20 | 4 | 96 | 12344.49 | 8.18 |
| SinsLTP8 | Seita.3G157400 | 101 | 31 | 70 | 19 | 10 | 7 | 34 | 7382.74 | 9.47 |
| SinsLTP9 | Seita.3G157500 | 95 | 27 | 68 | 37 | 9 | 5 | 44 | 7108.27 | 8.72 |
| SinsLTP10 | Seita.3G204700 | 132 | 39 | 93 | 64 | 11 | 4 | 53 | 9535.11 | 9.41 |
| SinsLTP11 | Seita.4G174500 | 120 | 28 | 92 | 39 | 11 | 2 | 40 | 9685.92 | 4.29 |
| SinsLTP12 | Seita.4G174600 | 116 | 24 | 92 | 59 | 7 | 0 | 50 | 9724.92 | 4.29 |
| SinsLTP13 | Seita.4G272200 | 94 | 27 | 67 | 34 | 7 | 5 | 48 | 6919.11 | 9.05 |
| SinsLTP14 | Seita.5G019900 | 132 | 30 | 102 | 60 | 14 | 8 | 50 | 10156.77 | 4.44 |
| SinsLTP15 | Seita.5G037500 | 114 | 23 | 91 | 48 | 11 | 1 | 54 | 9313.71 | 8.21 |
| SinsLTP16 | Seita.5G278800 | 101 | 27 | 74 | 37 | 9 | 8 | 47 | 8250.76 | 10.25 |
| SinsLTP17 | Seita.5G278900 | 96 | 22 | 74 | 39 | 13 | 6 | 38 | 7993.37 | 9.25 |
| SinsLTP18 | Seita.5G349800 | 125 | 22 | 103 | 35 | 10 | 1 | 57 | 10902.50 | 6.25 |
| SinsLTP19 | Seita.5G349900 | 109 | 24 | 85 | 53 | 15 | 5 | 36 | 9353.01 | 8.13 |
| SinsLTP20 | Seita.5G350000 | 109 | 24 | 85 | 57 | 12 | 6 | 34 | 9114.84 | 8.44 |
| SinsLTP21 | Seita.5G363000 | 124 | 28 | 96 | 38 | 12 | 2 | 44 | 10072.70 | 8.91 |
| SinsLTP22 | Seita.5G381800 | 118 | 27 | 91 | 56 | 6 | 3 | 53 | 9460.30 | 9.79 |
| SinsLTP23 | Seita.5G425600 | 105 | 25 | 80 | 26 | 10 | 0 | 44 | 8424.71 | 8.09 |
| SinsLTP24 | Seita.6G033000 | 111 | 26 | 85 | 77 | 6 | 5 | 23 | 9336.86 | 6.12 |
| SinsLTP25 | Seita.6G074400 | 123 | 30 | 93 | 59 | 9 | 3 | 52 | 9760.31 | 8.21 |
| SinsLTP26 | Seita.7G093400 | 112 | 18 | 94 | 31 | 6 | 3 | 54 | 9753.39 | 8.83 |
| SinsLTP27 | Seita.7G093500 | 114 | 22 | 92 | 45 | 11 | 4 | 54 | 9367.89 | 9.08 |
| SinsLTP28 | Seita.7G093600 | 118 | 15 | 103 | 38 | 11 | 3 | 66 | 10630.16 | 7.06 |
| SinsLTP29 | Seita.7G238500 | 126 | 27 | 99 | 61 | 9 | 4 | 52 | 10872.77 | 8.85 |
| SinsLTP30 | Seita.7G300900 | 121 | 29 | 92 | 52 | 12 | 3 | 54 | 9436.95 | 8.70 |
| SinsLTP31 | Seita.7G301000 | 120 | 25 | 95 | 36 | 12 | 2 | 45 | 9056.24 | 8.52 |
| SinsLTP32 | Seita.7G301100 | 117 | 24 | 93 | 67 | 10 | 0 | 40 | 9270.44 | 9.25 |
| SinsLTP33 | Seita.7G301200 | 121 | 28 | 93 | 64 | 8 | 1 | 48 | 9351.91 | 9.41 |
| SinsLTP34 | Seita.8G013500 | 121 | 28 | 93 | 59 | 8 | 2 | 52 | 9237.80 | 9.38 |
| SinsLTP35 | Seita.8G013600 | 117 | 24 | 93 | 56 | 13 | 2 | 46 | 9196.32 | 9.30 |
| SinsLTP36 | Seita.8G013700 | 120 | 25 | 95 | 35 | 12 | 2 | 46 | 9188.46 | 8.74 |
| SinsLTP37 | Seita.8G013800 | 121 | 29 | 92 | 52 | 16 | 2 | 51 | 9423.95 | 8.70 |
| SinsLTP38 | Seita.9G130000 | 110 | 27 | 83 | 41 | 17 | 4 | 48 | 8707.03 | 9.68 |
| SinsLTP39 | Seita.9G199200 | 100 | 23 | 77 | 46 | 9 | 5 | 40 | 8278.46 | 7.71 |
| SinsLTP40 | Seita.9G199300 | 95 | 25 | 70 | 32 | 12 | 7 | 44 | 7688.64 | 4.99 |
| SinsLTP41 | Seita.9G199600 | 93 | 25 | 68 | 35 | 14 | 10 | 34 | 6947.92 | 7.71 |
| SinsLTP42 | Seita.9G287800 | 117 | 26 | 91 | 55 | 8 | 7 | 47 | 10040.38 | 8.16 |
| SinsLTP43 | Seita.9G288000 | 114 | 29 | 85 | 59 | 10 | 6 | 39 | 9180.38 | 5.76 |
| SinsLTP44 | Seita.9G382100 | 126 | 26 | 100 | 37 | 9 | 1 | 53 | 9919.32 | 6.24 |
| SinsLTP45 | Seita.9G570900 | 93 | 25 | 68 | 40 | 9 | 7 | 37 | 6906.95 | 9.07 |
