# Supplementary material for: Genome-wide identification, molecular evolution and expression analysis of the non-specific lipid transfer protein (nsLTP) family in Setaria italica
Source: BMC Plant Biol. 2022 Nov 28;22:547. doi: 10.1186/s12870-022-03921-1 (PMC9703814; doi:10.1186/s12870-022-03921-1)
Supplement: Supplementary file 2 — Additional file 2. The nsLTPs identified in S. viridis, S. bicolor, Z. mays, O. sativa and B. distachyon in this study. [file 12870_2022_3921_MOESM2_ESM.docx]

**Additional file 2:** The *nsLTPs* identified in *S. viridis*, *S. bicolor*, *Z. mays*, *O. sativa* and *B. distachyon* in this study

| Name | Locus Name | Subfamily | Chromosome Name | Gene Start (bp) | Gene End (bp) |
| --- | --- | --- | --- | --- | --- |
| *SvnsLTP1* | Sevir.2G082400 | Type VI | Chr_02 | 7055063 | 7055575 |
| *SvnsLTP2* | Sevir.2G112300 | Type IV | Chr_02 | 11001099 | 11001832 |
| *SvnsLTP3* | Sevir.2G112600 | Type IV | Chr_02 | 11039896 | 11040620 |
| *SvnsLTP4* | Sevir.2G112700 | Type IV | Chr_02 | 11042753 | 11043410 |
| *SvnsLTP5* | Sevir.2G112800 | Type IV | Chr_02 | 11046667 | 11047361 |
| *SvnsLTP6* | Sevir.2G112900 | Type IV | Chr_02 | 11049658 | 11050368 |
| *SvnsLTP7* | Sevir.2G429500 | Single | Chr_02 | 46092861 | 46093639 |
| *SvnsLTP8* | Sevir.3G160500 | Type II | Chr_03 | 11352921 | 11353837 |
| *SvnsLTP9* | Sevir.3G160600 | Type II | Chr_03 | 11356063 | 11356729 |
| *SvnsLTP10* | Sevir.3G209600 | Type I | Chr_03 | 15522834 | 15525051 |
| *SvnsLTP11* | Sevir.4G142700 | Type I | Chr_04 | 18467668 | 18468633 |
| *SvnsLTP12* | Sevir.4G142800 | Type I | Chr_04 | 18470637 | 18471387 |
| *SvnsLTP13* | Sevir.4G268500 | Type II | Chr_04 | 37009560 | 37014988 |
| *SvnsLTP14* | Sevir.4G284500 | Type II | Chr_04 | 38234489 | 38235308 |
| *SvnsLTP15* | Sevir.5G018500 | Type I | Chr_05 | 1259619 | 1260507 |
| *SvnsLTP16* | Sevir.5G035500 | Type V | Chr_05 | 2649704 | 2651015 |
| *SvnsLTP17* | Sevir.5G281300 | Type II | Chr_05 | 32809662 | 32810239 |
| *SvnsLTP18* | Sevir.5G281400 | Type II | Chr_05 | 32811585 | 32812242 |
| *SvnsLTP19* | Sevir.5G354600 | Type VI | Chr_05 | 38154170 | 38155325 |
| *SvnsLTP20* | Sevir.5G354700 | Type VI | Chr_05 | 38155866 | 38157445 |
| *SvnsLTP21* | Sevir.5G354800 | Type VI | Chr_05 | 38157883 | 38158490 |
| *SvnsLTP22* | Sevir.5G387100 | Type V | Chr_05 | 40304813 | 40307081 |
| *SvnsLTP23* | Sevir.5G431200 | Type IV | Chr_05 | 43372573 | 43373247 |
| *SvnsLTP24* | Sevir.6G073500 | Type I | Chr_06 | 6534060 | 6534967 |
| *SvnsLTP25* | Sevir.7G093100 | Type II | Chr_07 | 17951310 | 17951600 |
| *SvnsLTP26* | Sevir.7G101000 | Type V | Chr_07 | 18515589 | 18516493 |
| *SvnsLTP27* | Sevir.7G101100 | Type V | Chr_07 | 18519004 | 18519928 |
| *SvnsLTP28* | Sevir.7G101300 | Type V | Chr_07 | 18543481 | 18543928 |
| *SvnsLTP29* | Sevir.7G250000 | Type VI | Chr_07 | 29066830 | 29067309 |
| *SvnsLTP30* | Sevir.7G312100 | Type I | Chr_07 | 33253390 | 33254306 |
| *SvnsLTP31* | Sevir.7G312200 | Type I | Chr_07 | 33256403 | 33257903 |
| *SvnsLTP32* | Sevir.7G312300 | Type I | Chr_07 | 33261807 | 33262625 |
| *SvnsLTP33* | Sevir.7G312400 | Type I | Chr_07 | 33267306 | 33268250 |
| *SvnsLTP34* | Sevir.8G012400 | Type I | Chr_08 | 801710 | 802662 |
| *SvnsLTP35* | Sevir.8G012600 | Type I | Chr_08 | 810684 | 811551 |
| *SvnsLTP36* | Sevir.8G012700 | Type I | Chr_08 | 813704 | 814681 |
| *SvnsLTP37* | Sevir.9G058000 | Single | Chr_09 | 3395828 | 3397023 |
| *SvnsLTP38* | Sevir.9G198500 | Type II | Chr_09 | 14025345 | 14025985 |
| *SvnsLTP39* | Sevir.9G198600 | Type II | Chr_09 | 14053453 | 14054350 |
| *SvnsLTP40* | Sevir.9G198900 | Type II | Chr_09 | 14071331 | 14072020 |
| *SvnsLTP41* | Sevir.9G292000 | Type VI | Chr_09 | 30875463 | 30876668 |
| *SvnsLTP42* | Sevir.9G292200 | Type VI | Chr_09 | 30915308 | 30916160 |
| *SvnsLTP43* | Sevir.9G295800 | Type VI | Chr_09 | 31512755 | 31513864 |
| *SvnsLTP44* | Sevir.9G387100 | Single | Chr_09 | 42230816 | 42231373 |
| *SvnsLTP45* | Sevir.9G574100 | Type II | Chr_09 | 55481178 | 55481956 |
| *SbnsLTP1* | Sobic.001G200800 | Type II | Chr_01 | 18191249 | 18192020 |
| *SbnsLTP2* | Sobic.001G200900 | Type II | Chr_01 | 18194042 | 18194584 |
| *SbnsLTP3* | Sobic.001G266500 | Type VI | Chr_01 | 50786217 | 50787463 |
| *SbnsLTP4* | Sobic.001G266900 | Type VI | Chr_01 | 50852244 | 50852758 |
| *SbnsLTP5* | Sobic.002G109100 | Type IV | Chr_02 | 13271051 | 13272067 |
| *SbnsLTP6* | Sobic.002G109400 | Type IV | Chr_02 | 13300631 | 13301496 |
| *SbnsLTP7* | Sobic.002G109500 | Type IV | Chr_02 | 13337493 | 13338174 |
| *SbnsLTP8* | Sobic.003G016000 | Type I | Chr_03 | 1451110 | 1452311 |
| *SbnsLTP9* | Sobic.003G263800 | Type II | Chr_03 | 60113010 | 60113303 |
| *SbnsLTP10* | Sobic.003G326200 | Type VI | Chr_03 | 65225249 | 65226065 |
| *SbnsLTP11* | Sobic.003G326300 | Type VI | Chr_03 | 65232773 | 65233430 |
| *SbnsLTP12* | Sobic.003G338700 | Type I | Chr_03 | 66179184 | 66180033 |
| *SbnsLTP13* | Sobic.003G356500 | Type V | Chr_03 | 67490999 | 67492096 |
| *SbnsLTP14* | Sobic.003G399600 | Type IV | Chr_03 | 70856724 | 70857418 |
| *SbnsLTP15* | Sobic.003G399700 | Type IV | Chr_03 | 70860039 | 70860356 |
| *SbnsLTP16* | Sobic.005G012600 | Type I | Chr_05 | 1148568 | 1148907 |
| *SbnsLTP17* | Sobic.005G096101 | Type V | Chr_05 | 15112039 | 15113474 |
| *SbnsLTP18* | Sobic.005G110000 | Type II | Chr_05 | 23955352 | 23955790 |
| *SbnsLTP19* | Sobic.005G110040 | Type II | Chr_05 | 23961791 | 23962226 |
| *SbnsLTP20* | Sobic.006G074200 | Type V | Chr_06 | 43649937 | 43650733 |
| *SbnsLTP21* | Sobic.006G074300 | Type V | Chr_06 | 43654083 | 43654900 |
| *SbnsLTP22* | Sobic.007G001600 | Type II | Chr_07 | 135237 | 135640 |
| *SbnsLTP23* | Sobic.007G180700 | Type III | Chr_07 | 61403378 | 61404684 |
| *SbnsLTP24* | Sobic.008G030700 | Type I | Chr_08 | 2745376 | 2746436 |
| *SbnsLTP25* | Sobic.008G030900 | Type I | Chr_08 | 2751929 | 2760254 |
| *SbnsLTP26* | Sobic.008G031000 | Type I | Chr_08 | 2778882 | 2780069 |
| *SbnsLTP27* | Sobic.008G063800 | Type VI | Chr_08 | 6912515 | 6912956 |
| *SbnsLTP28* | Sobic.008G064000 | Type VI | Chr_08 | 6996069 | 6996753 |
| *SbnsLTP29* | Sobic.009G221900 | Type II | Chr_09 | 56488517 | 56490066 |
| *SbnsLTP30* | Sobic.009G222000 | Type II | Chr_09 | 56491188 | 56492179 |
| *SbnsLTP31* | Sobic.010G159500 | Type I | Chr_10 | 46845559 | 46846569 |
| *SbnsLTP32* | Sobic.K027700 | Type II | super_2843 | 2351 | 2626 |
| *ZmnsLTP1* | Zm00008a000084 | Type II | Chr_01 | 3083701 | 3083997 |
| *ZmnsLTP2* | Zm00008a007422 | Type V | Chr_02 | 53289836 | 53290748 |
| *ZmnsLTP3* | Zm00008a008118 | Type V | Chr_02 | 132189661 | 132190620 |
| *ZmnsLTP4* | Zm00008a008463 | Type IV | Chr_02 | 162413154 | 162413501 |
| *ZmnsLTP5* | Zm00008a010055 | Type II | Chr_02 | 224617500 | 224617805 |
| *ZmnsLTP6* | Zm00008a012959 | Type IV | Chr_03 | 162543163 | 162543483 |
| *ZmnsLTP7* | Zm00008a014788 | Type I | Chr_03 | 231371296 | 231372507 |
| *ZmnsLTP8* | Zm00008a014816 | Type I | Chr_03 | 232336769 | 232337237 |
| *ZmnsLTP9* | Zm00008a019923 | Type II | Chr_05 | 22905075 | 22905362 |
| *ZmnsLTP10* | Zm00008a020236 | Type VI | Chr_05 | 38862080 | 38863307 |
| *ZmnsLTP11* | Zm00008a024462 | Type II | Chr_06 | 85733641 | 85733925 |
| *ZmnsLTP12* | Zm00008a026362 | Type II | Chr_06 | 158924228 | 158924524 |
| *ZmnsLTP13* | Zm00008a026904 | Single | Chr_07 | 9207899 | 9208297 |
| *ZmnsLTP14* | Zm00008a027041 | Type VII | Chr_07 | 15606324 | 15606692 |
| *ZmnsLTP15* | Zm00008a027257 | Type IV | Chr_07 | 29304515 | 29304826 |
| *ZmnsLTP16* | Zm00008a027258 | Type IV | Chr_07 | 29433834 | 29434151 |
| *ZmnsLTP17* | Zm00008a028618 | Type III | Chr_07 | 141438135 | 141438859 |
| *ZmnsLTP18* | Zm00008a032304 | Type II | Chr_08 | 152439825 | 152440127 |
| *ZmnsLTP19* | Zm00008a034321 | Type I | Chr_09 | 65778534 | 65778887 |
| *ZmnsLTP20* | Zm00008a036396 | Type I | Chr_10 | 4167290 | 4168192 |
| *LOC_Os01g12020.1* | LOC_Os01g12020 | Type I | Chr_1 | 6541005 | 6543076 |
| *LOC_Os01g49640.1* | LOC_Os01g49640 | Type II | Chr_1 | 28537873 | 28538617 |
| *LOC_Os01g49650.1* | LOC_Os01g49650 | Type II | Chr_1 | 28538774 | 28539704 |
| *LOC_Os01g58650.1* | LOC_Os01g58650 | Type VI | Chr_1 | 33906370 | 33909592 |
| *LOC_Os01g58660.1* | LOC_Os01g58660 | Type VI | Chr_1 | 33911298 | 33911989 |
| *LOC_Os01g60740.1* | LOC_Os01g60740 | Type I | Chr_1 | 35129858 | 35130917 |
| *LOC_Os01g62980.1* | LOC_Os01g62980 | Type V | Chr_1 | 36483566 | 36484818 |
| *LOC_Os01g62980.2* | LOC_Os01g62980 | Type V | Chr_1 | 36483566 | 36484818 |
| *LOC_Os01g68580.1* | LOC_Os01g68580 | Type IV | Chr_1 | 39836639 | 39837257 |
| *LOC_Os01g68589.1* | LOC_Os01g68589 | Type IV | Chr_1 | 39839215 | 39840447 |
| *LOC_Os03g02050.1* | LOC_Os03g02050 | Type II | Chr_3 | 651993 | 652803 |
| *LOC_Os03g14630.1* | LOC_Os03g14630 | Single | Chr_3 | 7946467 | 7947180 |
| *LOC_Os03g25350.1* | LOC_Os03g25350 | Type VI | Chr_3 | 14491736 | 14492430 |
| *LOC_Os03g44000.1* | LOC_Os03g44000 | Single | Chr_3 | 24721228 | 24723141 |
| *LOC_Os03g59380.1* | LOC_Os03g59380 | Type I | Chr_3 | 33798940 | 33799765 |
| *LOC_Os04g33920.1* | LOC_Os04g33920 | Type V | Chr_4 | 20543121 | 20544485 |
| *LOC_Os05g06780.1* | LOC_Os05g06780 | Type V | Chr_5 | 3539563 | 3540972 |
| *LOC_Os05g40010.1* | LOC_Os05g40010 | Type I | Chr_5 | 23508004 | 23509669 |
| *LOC_Os05g47700.1* | LOC_Os05g47700 | Type II | Chr_5 | 27330644 | 27331350 |
| *LOC_Os05g47730.1* | LOC_Os05g47730 | Type II | Chr_5 | 27345715 | 27346293 |
| *LOC_Os06g06340.1* | LOC_Os06g06340 | Type I | Chr_6 | 2947711 | 2948191 |
| *LOC_Os06g34840.1* | LOC_Os06g34840 | Type I | Chr_6 | 20254443 | 20257626 |
| *LOC_Os06g49190.1* | LOC_Os06g49190 | Type II | Chr_6 | 29807815 | 29808464 |
| *LOC_Os07g18990.1* | LOC_Os07g18990 | Type IV | Chr_7 | 11241309 | 11242149 |
| *LOC_Os07g27940.1* | LOC_Os07g27940 | Type I | Chr_7 | 16294810 | 16295362 |
| *LOC_Os10g05720.1* | LOC_Os10g05720 | Type VI | Chr_10 | 2885293 | 2886579 |
| *LOC_Os10g05720.2* | LOC_Os10g05720 | Type VI | Chr_10 | 2885293 | 2886579 |
| *LOC_Os10g36070.1* | LOC_Os10g36070 | Type II | Chr_10 | 19288018 | 19288320 |
| *LOC_Os10g36090.1* | LOC_Os10g36090 | Type II | Chr_10 | 19299589 | 19299891 |
| *LOC_Os10g36100.1* | LOC_Os10g36100 | Type II | Chr_10 | 19303783 | 19304347 |
| *LOC_Os10g36110.1* | LOC_Os10g36110 | Type II | Chr_10 | 19307415 | 19308081 |
| *LOC_Os10g36160.1* | LOC_Os10g36160 | Type II | Chr_10 | 19330372 | 19330978 |
| *LOC_Os10g36170.1* | LOC_Os10g36170 | Type II | Chr_10 | 19335022 | 19335615 |
| *LOC_Os11g02369.1* | LOC_Os11g02369 | Type I | Chr_11 | 693419 | 693864 |
| *LOC_Os11g02389.1* | LOC_Os11g02389 | Type I | Chr_11 | 703048 | 703960 |
| *LOC_Os11g02400.1* | LOC_Os11g02400 | Type I | Chr_11 | 715400 | 716542 |
| *LOC_Os11g02424.2* | LOC_Os11g02424 | Type I | Chr_11 | 722930 | 726974 |
| *LOC_Os11g24070.1* | LOC_Os11g24070 | Type I | Chr_11 | 13668517 | 13669643 |
| *LOC_Os11g29420.1* | LOC_Os11g29420 | Type VI | Chr_11 | 17080223 | 17081391 |
| *LOC_Os11g37280.1* | LOC_Os11g37280 | Type VII | Chr_11 | 22016848 | 22018539 |
| *LOC_Os12g02310.1* | LOC_Os12g02310 | Type I | Chr_12 | 732978 | 734220 |
| *LOC_Os12g02310.2* | LOC_Os12g02310 | Type I | Chr_12 | 732978 | 734220 |
| *LOC_Os12g02320.1* | LOC_Os12g02320 | Type I | Chr_12 | 737406 | 737894 |
| *LOC_Os12g02330.2* | LOC_Os12g02330 | Type I | Chr_12 | 746155 | 747300 |
| *LOC_Os12g02340.1* | LOC_Os12g02340 | Type I | Chr_12 | 760919 | 762534 |
| *BdnsLTP1* | Bradi1g04430 | Type I | Chr_1 | 2990367 | 2991345 |
| *BdnsLTP2* | Bradi1g19470 | Type IV | Chr_1 | 15575926 | 15576237 |
| *BdnsLTP3* | Bradi1g21870 | Type I | Chr_1 | 17587330 | 17588274 |
| *BdnsLTP4* | Bradi1g28422 | Type I | Chr_1 | 23690764 | 23691708 |
| *BdnsLTP5* | Bradi1g39120 | Type I | Chr_1 | 35783855 | 35785060 |
| *BdnsLTP6* | Bradi1g48790 | Type I | Chr_1 | 47615691 | 47617584 |
| *BdnsLTP7* | Bradi1g77820 | Type II | Chr_1 | 74223373 | 74223969 |
| *BdnsLTP8* | Bradi2g07140 | Type I | Chr_2 | 5488348 | 5489155 |
| *BdnsLTP9* | Bradi2g17530 | Type II | Chr_2 | 15613940 | 15614551 |
| *BdnsLTP10* | Bradi2g17540 | Type II | Chr_2 | 15616011 | 15616872 |
| *BdnsLTP11* | Bradi2g17550 | Type II | Chr_2 | 15618209 | 15618896 |
| *BdnsLTP12* | Bradi2g18590 | Type I | Chr_2 | 16504288 | 16505196 |
| *BdnsLTP13* | Bradi2g30490 | Type V | Chr_2 | 29899931 | 29900785 |
| *BdnsLTP14* | Bradi2g47072 | Type II | Chr_2 | 47202869 | 47203162 |
| *BdnsLTP15* | Bradi2g52520 | Type VI | Chr_2 | 51650809 | 51651806 |
| *BdnsLTP16* | Bradi2g52525 | Type VI | Chr_2 | 51654214 | 51654450 |
| *BdnsLTP17* | Bradi2g53570 | Type I | Chr_2 | 52501382 | 52502359 |
| *BdnsLTP18* | Bradi2g58520 | Type IV | Chr_2 | 56171020 | 56171818 |
| *BdnsLTP19* | Bradi2g58530 | Type IV | Chr_2 | 56173547 | 56174306 |
| *BdnsLTP20* | Bradi3g21070 | Type VI | Chr_3 | 20071878 | 20072996 |
| *BdnsLTP21* | Bradi3g30290 | Type II | Chr_3 | 32122361 | 32122645 |
| *BdnsLTP22* | Bradi3g30300 | Type II | Chr_3 | 32129887 | 32130589 |
| *BdnsLTP23* | Bradi3g30310 | Type II | Chr_3 | 32158547 | 32159483 |
| *BdnsLTP24* | Bradi3g58060 | Type IV | Chr_3 | 57385171 | 57387058 |
| *BdnsLTP25* | Bradi4g15400 | Type VI | Chr_4 | 16107752 | 16108423 |
| *BdnsLTP26* | Bradi4g25750 | Type I | Chr_4 | 30980818 | 30981657 |
| *BdnsLTP27* | Bradi4g44400 | Type I | Chr_4 | 47762722 | 47763829 |
| *BdnsLTP28* | Bradi4g44410 | Type I | Chr_4 | 47767234 | 47768040 |
| *BdnsLTP29* | Bradi5g09617 | Type V | Chr_5 | 13094567 | 13095351 |
| *BdnsLTP30* | Bradi5g22460 | Type VI | Chr_5 | 24985436 | 24985837 |
