# Supplementary material for: Genome-wide identification, molecular evolution and expression analysis of the non-specific lipid transfer protein (nsLTP) family in Setaria italica
Source: BMC Plant Biol. 2022 Nov 28;22:547. doi: 10.1186/s12870-022-03921-1 (PMC9703814; doi:10.1186/s12870-022-03921-1)
Supplement: Supplementary file 3 — Additional file 3. The classification and gene structures of nsLTPs in S. italica. [file 12870_2022_3921_MOESM3_ESM.docx]

**Additional file 3:** The classification and gene structures of *nsLTPs* in *S. italica*

| Name | Subfamily | Exon number | Intron number |
| --- | --- | --- | --- |
| *SinsLTP1* | Type VI | 2 | 1 |
| *SinsLTP2* | Type IV | 1 | 0 |
| *SinsLTP3* | Type IV | 1 | 0 |
| *SinsLTP4* | Type IV | 1 | 0 |
| *SinsLTP5* | Type IV | 1 | 0 |
| *SinsLTP6* | Type IV | 1 | 0 |
| *SinsLTP7* | Single | 2 | 1 |
| *SinsLTP8* | Type II | 1 | 0 |
| *SinsLTP9* | Type II | 1 | 0 |
| *SinsLTP10* | Type I | 1 | 0 |
| *SinsLTP11* | Type I | 1 | 0 |
| *SinsLTP12* | Type I | 1 | 0 |
| *SinsLTP13* | Type II | 1 | 0 |
| *SinsLTP14* | Type I | 2 | 1 |
| *SinsLTP15* | Type V | 2 | 1 |
| *SinsLTP16* | Type II | 1 | 0 |
| *SinsLTP17* | Type II | 1 | 0 |
| *SinsLTP18* | Type VI | 2 | 1 |
| *SinsLTP19* | Type VI | 2 | 1 |
| *SinsLTP20* | Type VI | 2 | 1 |
| *SinsLTP21* | Type I | 1 | 0 |
| *SinsLTP22* | Type V | 2 | 1 |
| *SinsLTP23* | Type IV | 1 | 0 |
| *SinsLTP24* | Type VI | 2 | 1 |
| *SinsLTP25* | Type I | 1 | 0 |
| *SinsLTP26* | Type V | 2 | 1 |
| *SinsLTP27* | Type V | 2 | 1 |
| *SinsLTP28* | Type V | 2 | 1 |
| *SinsLTP29* | Type VI | 2 | 1 |
| *SinsLTP30* | Type I | 1 | 0 |
| *SinsLTP31* | Type I | 1 | 0 |
| *SinsLTP32* | Type I | 1 | 0 |
| *SinsLTP33* | Type I | 1 | 0 |
| *SinsLTP34* | Type I | 1 | 0 |
| *SinsLTP35* | Type I | 1 | 0 |
| *SinsLTP36* | Type I | 1 | 0 |
| *SinsLTP37* | Type I | 1 | 0 |
| *SinsLTP38* | Single | 1 | 0 |
| *SinsLTP39* | Type II | 1 | 0 |
| *SinsLTP40* | Type II | 1 | 0 |
| *SinsLTP41* | Type II | 1 | 0 |
| *SinsLTP42* | Type VI | 2 | 1 |
| *SinsLTP43* | Type VI | 2 | 1 |
| *SinsLTP44* | Single | 2 | 1 |
| *SinsLTP45* | Type II | 1 | 0 |
