# Supplementary material for: Genome-wide identification, molecular evolution and expression analysis of the non-specific lipid transfer protein (nsLTP) family in Setaria italica
Source: BMC Plant Biol. 2022 Nov 28;22:547. doi: 10.1186/s12870-022-03921-1 (PMC9703814; doi:10.1186/s12870-022-03921-1)
Supplement: Supplementary file 4 — Additional file 4. The percentage of members in each nsLTP subfamily in S. italica (a), S. viridis (b), S. bicolor (c), Z. mays (d), O. sativa (e) and B. distachyon (f). [file 12870_2022_3921_MOESM4_ESM.docx]

**
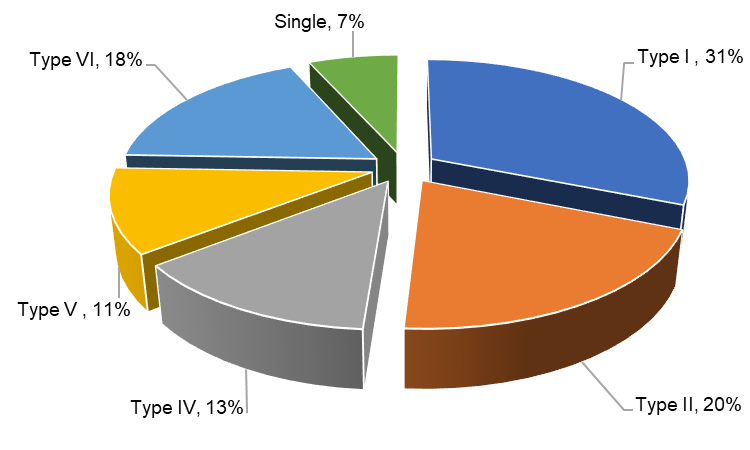

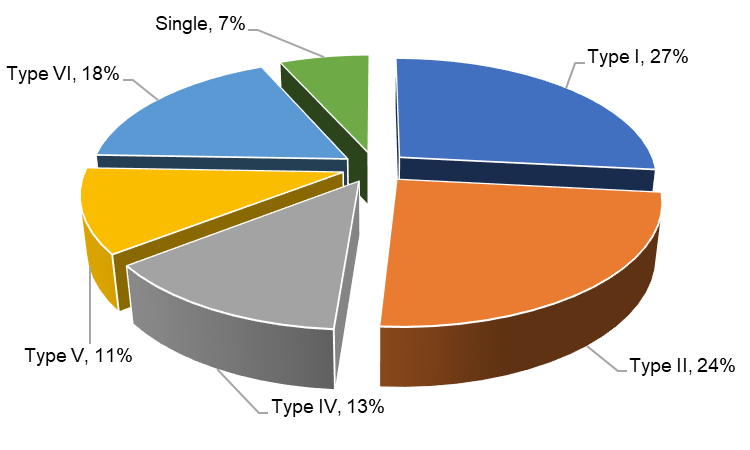
a b**


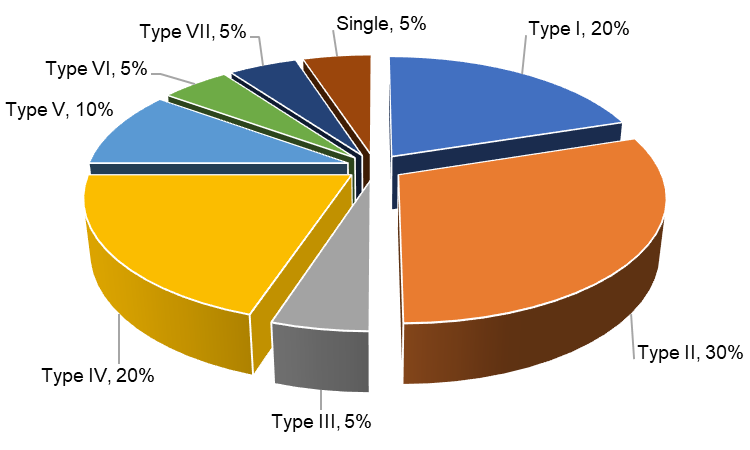
**
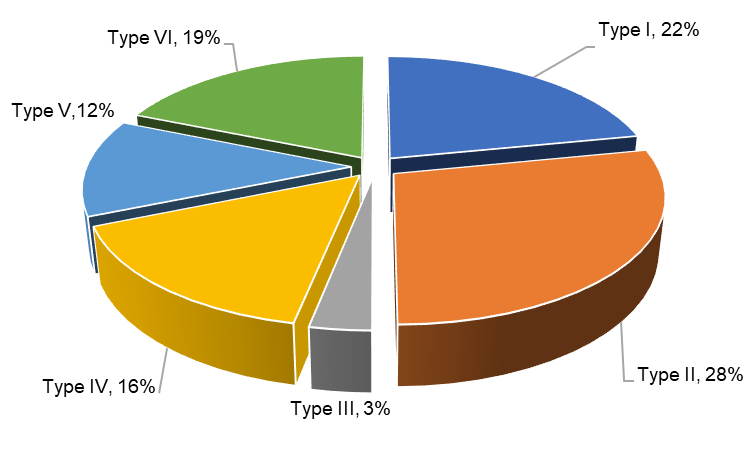
c d**


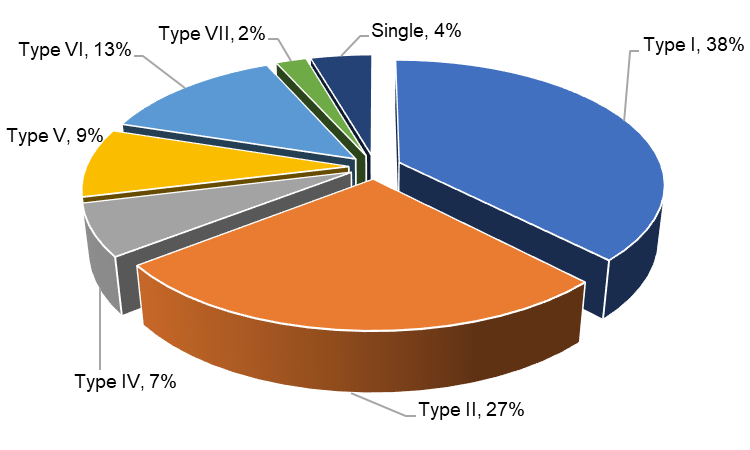

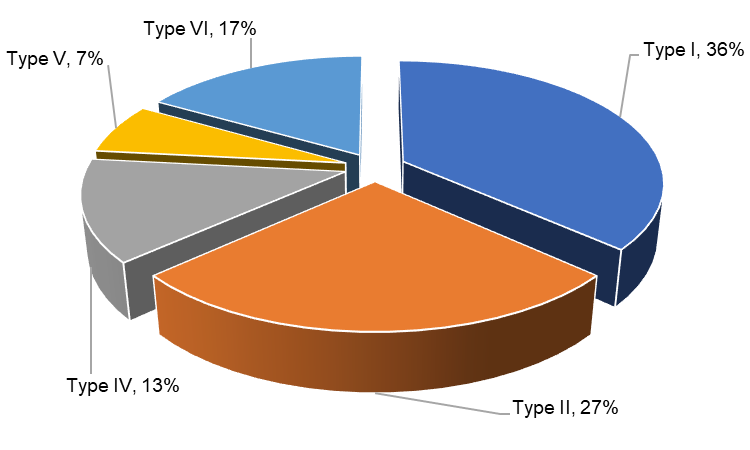
**e f**

**Additional file 4:** The percentage of members in each *nsLTP* subfamily in *S. italica* (**a**), *S. viridis* (**b**), *S. bicolor* (**c**), *Z. mays* (**d**), *O. sativa* (**e**) and *B. distachyon* (**f**).
