# Supplementary material for: Genome-wide identification, molecular evolution and expression analysis of the non-specific lipid transfer protein (nsLTP) family in Setaria italica
Source: BMC Plant Biol. 2022 Nov 28;22:547. doi: 10.1186/s12870-022-03921-1 (PMC9703814; doi:10.1186/s12870-022-03921-1)
Supplement: Supplementary file 5 — Additional file 5. Phylogenetic relationships of the nsLTPs in S. italica, S. viridis, S. bicolor, Z. mays, O. sativa, B. distachyon and Arabidopsis. Amino acid sequences were aligned using ClustalW and the neighbor-joining tree was generated through the MEGA7 program. The subfamilies are labeled and denoted by different colors and the numbers at nodes represent bootstrap support values from 1000 replicates. [file 12870_2022_3921_MOESM5_ESM.docx]

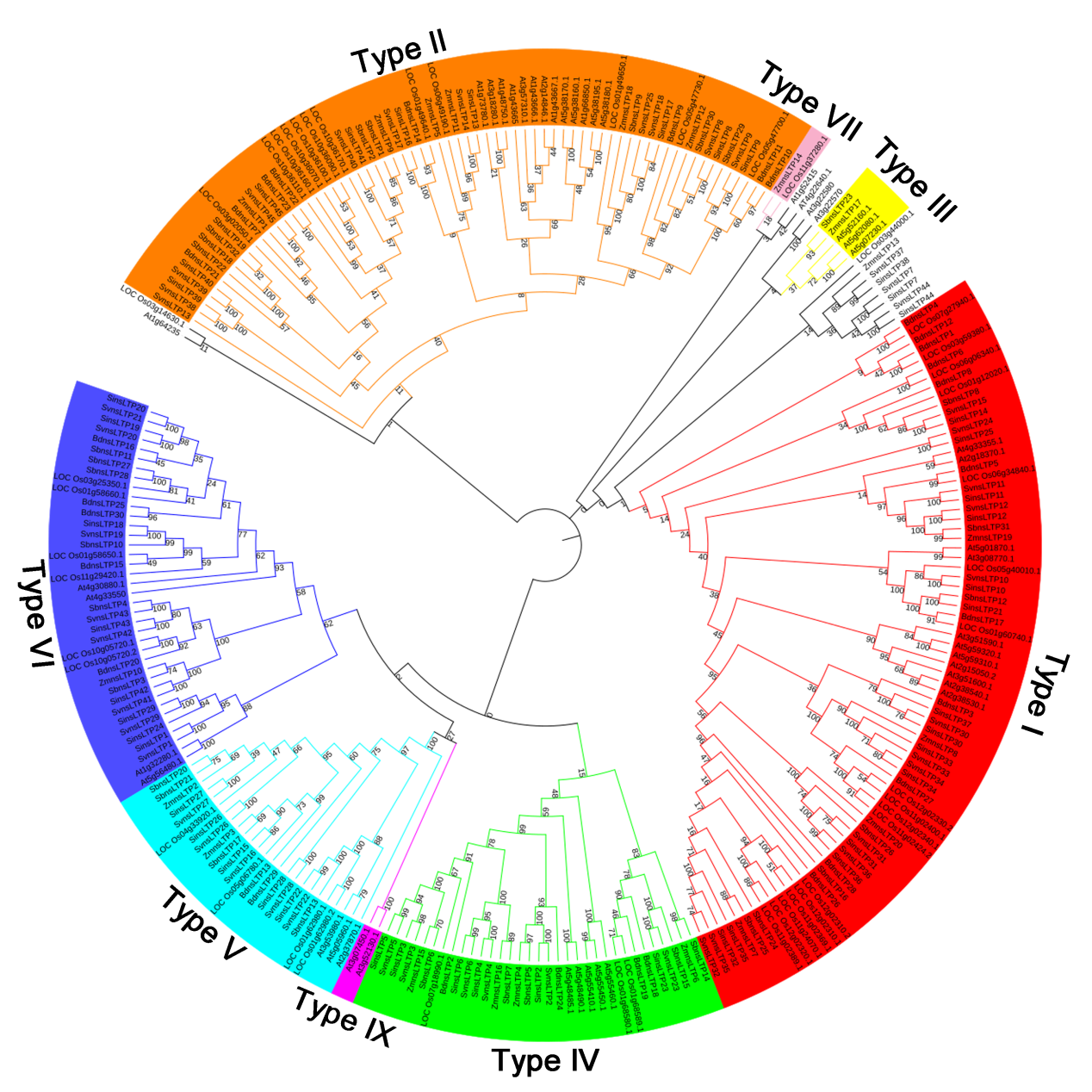


**Additional file 5:** Phylogenetic relationships of the nsLTPs in *S. italica*, *S. viridis*, *S. bicolor*, *Z. mays*, *O. sativa*, *B. distachyon* and Arabidopsis. Amino acid sequences were aligned using ClustalW and the neighbor-joining tree was generated through the MEGA7 program. The subfamilies are labeled and denoted by different colors and the numbers at nodes represent bootstrap support values from 1000 replicates.
