# Supplementary material for: Genome-wide identification, molecular evolution and expression analysis of the non-specific lipid transfer protein (nsLTP) family in Setaria italica
Source: BMC Plant Biol. 2022 Nov 28;22:547. doi: 10.1186/s12870-022-03921-1 (PMC9703814; doi:10.1186/s12870-022-03921-1)
Supplement: Supplementary file 6 — Additional file 6. Genomic locations of nsLTPs in S. italica. [file 12870_2022_3921_MOESM6_ESM.docx]

**Additional file 6:** Genomic locations of *nsLTPs* in *S. italica*

| Name | Chromosome Name | Gene Start (bp) | Gene End (bp) |
| --- | --- | --- | --- |
| *SinsLTP1* | Chr_2 | 6921044 | 6921771 |
| *SinsLTP2* | Chr_2 | 10278425 | 10279640 |
| *SinsLTP3* | Chr_2 | 10325376 | 10326101 |
| *SinsLTP4* | Chr_2 | 10328254 | 10328862 |
| *SinsLTP5* | Chr_2 | 10332084 | 10332724 |
| *SinsLTP6* | Chr_2 | 10346138 | 10346836 |
| *SinsLTP7* | Chr_2 | 47311363 | 47312205 |
| *SinsLTP8* | Chr_3 | 11429249 | 11429554 |
| *SinsLTP9* | Chr_3 | 11433390 | 11433677 |
| *SinsLTP10* | Chr_3 | 15750256 | 15751942 |
| *SinsLTP11* | Chr_4 | 27974746 | 27975655 |
| *SinsLTP12* | Chr_4 | 27978021 | 27978778 |
| *SinsLTP13* | Chr_4 | 38836260 | 38837040 |
| *SinsLTP14* | Chr_5 | 2143360 | 2144253 |
| *SinsLTP15* | Chr_5 | 3470113 | 3470840 |
| *SinsLTP16* | Chr_5 | 33922336 | 33922641 |
| *SinsLTP17* | Chr_5 | 33924255 | 33924545 |
| *SinsLTP18* | Chr_5 | 39262374 | 39263201 |
| *SinsLTP19* | Chr_5 | 39263997 | 39264681 |
| *SinsLTP20* | Chr_5 | 39266072 | 39266758 |
| *SinsLTP21* | Chr_5 | 40129894 | 40130802 |
| *SinsLTP22* | Chr_5 | 41471821 | 41473872 |
| *SinsLTP23* | Chr_5 | 44577563 | 44577880 |
| *SinsLTP24* | Chr_6 | 2467228 | 2467707 |
| *SinsLTP25* | Chr_6 | 6575119 | 6576053 |
| *SinsLTP26* | Chr_7 | 19634568 | 19635451 |
| *SinsLTP27* | Chr_7 | 19637932 | 19638759 |
| *SinsLTP28* | Chr_7 | 19654149 | 19654596 |
| *SinsLTP29* | Chr_7 | 30070353 | 30070968 |
| *SinsLTP30* | Chr_7 | 34122693 | 34123587 |
| *SinsLTP31* | Chr_7 | 34125573 | 34126987 |
| *SinsLTP32* | Chr_7 | 34131642 | 34132470 |
| *SinsLTP33* | Chr_7 | 34137392 | 34138327 |
| *SinsLTP34* | Chr_8 | 790703 | 791681 |
| *SinsLTP35* | Chr_8 | 800674 | 801551 |
| *SinsLTP36* | Chr_8 | 803744 | 804625 |
| *SinsLTP37* | Chr_8 | 809687 | 810948 |
| *SinsLTP38* | Chr_9 | 8189583 | 8189915 |
| *SinsLTP39* | Chr_9 | 14247565 | 14248179 |
| *SinsLTP40* | Chr_9 | 14275212 | 14275830 |
| *SinsLTP41* | Chr_9 | 14292303 | 14292954 |
| *SinsLTP42* | Chr_9 | 32146324 | 32147617 |
| *SinsLTP43* | Chr_9 | 32194046 | 32194979 |
| *SinsLTP44* | Chr_9 | 44097607 | 44098703 |
| *SinsLTP45* | Chr_9 | 58263321 | 58264064 |
