# Supplementary material for: Genome-wide identification, molecular evolution and expression analysis of the non-specific lipid transfer protein (nsLTP) family in Setaria italica
Source: BMC Plant Biol. 2022 Nov 28;22:547. doi: 10.1186/s12870-022-03921-1 (PMC9703814; doi:10.1186/s12870-022-03921-1)
Supplement: Supplementary file 7 — Additional file 7. Ka/Ks analysis for orthologous nsLTP gene pairs between between S. italica and S. viridis, S. bicolor, Z. mays, O. sativa and B. distachyon. [file 12870_2022_3921_MOESM7_ESM.docx]

**Additional file 7:** Ka/Ks analysis for orthologous *nsLTP* gene pairs between between *S. italica* and *S. viridis*, *S. bicolor*, *Z. mays*, *O. sativa* and *B. distachyon*

| Orthologous gene pairs | | Subfamilies | Ka | Ks | Ka/Ks | Purifing selection |
| --- | --- | --- | --- | --- | --- | --- |
| *SinsLTP1* | *SvnsLTP1* | VI | 0.0156 | 0.0003 | 50.0000 | No |
| *SinsLTP2* | *SvnsLTP2* | IV | 0.0000 | 0.0316 | 0.0000 | Yes |
| *SinsLTP3* | *SvnsLTP3* | IV | 0.0000 | 0.0000 | 0.4489 | Yes |
| *SinsLTP4* | *SvnsLTP4* | IV | 0.0040 | 0.0736 | 0.0545 | Yes |
| *SinsLTP5* | *SvnsLTP5* | IV | 0.0000 | 0.0000 | 0.4360 | Yes |
| *SinsLTP6* | *SvnsLTP6* | IV | 0.0077 | 0.1061 | 0.0722 | Yes |
| *SinsLTP7* | *SvnsLTP7* | Single | 0.0000 | 0.0000 | 0.4275 | Yes |
| *SinsLTP8* | *SvnsLTP8* | II | 0.0000 | 0.0626 | 0.0000 | Yes |
| *SinsLTP9* | *SvnsLTP9* | II | 0.0000 | 0.0000 | 0.4402 | Yes |
| *SinsLTP10* | *SvnsLTP10* | I | 0.0000 | 0.0000 | 0.4860 | Yes |
| *SinsLTP11* | *SvnsLTP11* | I | 0.0032 | 0.0675 | 0.0478 | Yes |
| *SinsLTP12* | *SvnsLTP12* | I | 0.0068 | 0.0001 | 50.0000 | No |
| *SinsLTP13* | *SvnsLTP14* | II | 0.0086 | 0.0002 | 50.0000 | No |
| *SinsLTP14* | *SvnsLTP15* | I | 0.0093 | 0.0154 | 0.6086 | Yes |
| *SinsLTP15* | *SvnsLTP16* | V | 0.0214 | 0.0386 | 0.5548 | Yes |
| *SinsLTP16* | *SvnsLTP17* | II | 0.0041 | 0.0001 | 50.0000 | No |
| *SinsLTP17* | *SvnsLTP18* | II | 0.0078 | 0.0002 | 50.0000 | No |
| *SinsLTP18* | *SvnsLTP19* | VI | 0.0131 | 0.0003 | 50.0000 | No |
| *SinsLTP19* | *SvnsLTP20* | VI | 0.0195 | 0.0299 | 0.6509 | Yes |
| *SinsLTP20* | *SvnsLTP21* | VI | 0.0000 | 0.0000 | 0.4404 | Yes |
| *SinsLTP22* | *SvnsLTP22* | V | 0.0000 | 0.0000 | 0.4411 | Yes |
| *SinsLTP23* | *SvnsLTP23* | IV | 0.0000 | 0.0000 | 0.4151 | Yes |
| *SinsLTP25* | *SvnsLTP24* | I | 0.0219 | 0.0869 | 0.2519 | Yes |
| *SinsLTP26* | *SvnsLTP26* | V | 0.0000 | 0.0463 | 0.0000 | Yes |
| *SinsLTP27* | *SvnsLTP27* | V | 0.0000 | 0.0000 | 0.4407 | Yes |
| *SinsLTP28* | *SvnsLTP28* | V | 0.0000 | 0.0000 | 0.4562 | Yes |
| *SinsLTP29* | *SvnsLTP29* | VI | 0.0070 | 0.0001 | 50.0000 | No |
| *SinsLTP31* | *SvnsLTP31* | I | 0.0067 | 0.0206 | 0.3229 | Yes |
| *SinsLTP37* | *SvnsLTP30* | I | 0.0000 | 0.0000 | 0.4406 | Yes |
| *SinsLTP33* | *SvnsLTP33* | I | 0.0133 | 0.0003 | 50.0000 | No |
| *SinsLTP34* | *SvnsLTP34* | I | 0.0034 | 0.0001 | 50.0000 | No |
| *SinsLTP35* | *SvnsLTP35* | I | 0.0127 | 0.0384 | 0.3305 | Yes |
| *SinsLTP32* | *SvnsLTP32* | I | 0.0032 | 0.0001 | 50.0000 | No |
| *SinsLTP36* | *SvnsLTP36* | I | 0.0000 | 0.0000 | 0.4405 | Yes |
| *SinsLTP39* | *SvnsLTP38* | II | 0.0000 | 0.0000 | 0.4401 | Yes |
| *SinsLTP40* | *SvnsLTP39* | II | 0.0084 | 0.0002 | 50.0000 | No |
| *SinsLTP41* | *SvnsLTP40* | II | 0.0081 | 0.0421 | 0.1934 | Yes |
| *SinsLTP42* | *SvnsLTP41* | VI | 0.0000 | 0.0103 | 0.0000 | Yes |
| *SinsLTP43* | *SvnsLTP42* | VI | 0.0000 | 0.0130 | 0.0000 | Yes |
| *SinsLTP44* | *SvnsLTP44* | Single | 0.0108 | 0.0282 | 0.3822 | Yes |
| *SinsLTP45* | *SvnsLTP45* | II | 0.0080 | 0.0813 | 0.0983 | Yes |
| *SinsLTP2* | *SbnsLTP5* | IV | 0.1464 | 0.9923 | 0.1475 | Yes |
| *SinsLTP5* | *SbnsLTP6* | IV | 0.0313 | 0.4908 | 0.0637 | Yes |
| *SinsLTP6* | *SbnsLTP7* | IV | 0.0456 | 0.7993 | 0.0570 | Yes |
| *SinsLTP9* | *SbnsLTP29* | II | 0.0986 | 0.8394 | 0.1175 | Yes |
| *SinsLTP14* | *SbnsLTP8* | I | 0.1190 | 0.5008 | 0.2376 | Yes |
| *SinsLTP15* | *SbnsLTP17* | V | 0.0350 | 0.4752 | 0.0736 | Yes |
| *SinsLTP17* | *SbnsLTP9* | II | 0.0497 | 0.5139 | 0.0967 | Yes |
| *SinsLTP18* | *SbnsLTP10* | VI | 0.1105 | 0.3756 | 0.2941 | Yes |
| *SinsLTP21* | *SbnsLTP12* | I | 0.0640 | 0.4325 | 0.1479 | Yes |
| *SinsLTP22* | *SbnsLTP13* | V | 0.0168 | 0.5147 | 0.0326 | Yes |
| *SinsLTP27* | *SbnsLTP20* | V | 0.0931 | 0.2867 | 0.3248 | Yes |
| *SinsLTP35* | *SbnsLTP25* | I | 0.0723 | 9.0447 | 0.0080 | Yes |
| *SinsLTP36* | *SbnsLTP26* | I | 0.1084 | 1.1794 | 0.0919 | Yes |
| *SinsLTP2* | *ZmnsLTP4* | IV | 0.1183 | 1.0576 | 0.1118 | Yes |
| *SinsLTP3* | *ZmnsLTP15* | IV | 0.0443 | 0.6933 | 0.0639 | Yes |
| *SinsLTP4* | *ZmnsLTP16* | IV | 0.0676 | 1.1606 | 0.0583 | Yes |
| *SinsLTP8* | *ZmnsLTP12* | II | 0.0931 | 0.5518 | 0.1686 | Yes |
| *SinsLTP15* | *ZmnsLTP3* | V | 0.0423 | 0.6133 | 0.0689 | Yes |
| *SinsLTP17* | *ZmnsLTP18* | II | 0.0884 | 0.6250 | 0.1414 | Yes |
| *SinsLTP27* | *ZmnsLTP2* | V | 0.1207 | 0.2491 | 0.4844 | Yes |
| *SinsLTP34* | *ZmnsLTP8* | I | 0.1634 | 0.5317 | 0.3074 | Yes |
| *SinsLTP35* | *ZmnsLTP7* | I | 0.0913 | 1.3004 | 0.0702 | Yes |
| *SinsLTP36* | *ZmnsLTP20* | I | 0.1004 | 1.3912 | 0.0722 | Yes |
| *SinsLTP8* | *LOC_Os05g47730.1* | II | 0.1214 | 1.5415 | 0.0787 | Yes |
| *SinsLTP15* | *LOC_Os05g06780.1* | V | 0.0925 | 1.0917 | 0.0847 | Yes |
| *SinsLTP27* | *LOC_Os04g33920.1* | V | 0.1230 | 0.6572 | 0.1872 | Yes |
| *SinsLTP35* | *LOC_Os11g02369.1* | I | 0.1105 | 2.8390 | 0.0389 | Yes |
| *SinsLTP15* | *BdnsLTP13* | V | 0.0997 | 1.4469 | 0.0689 | Yes |
