# Supplementary material for: Genome-wide identification, molecular evolution and expression analysis of the non-specific lipid transfer protein (nsLTP) family in Setaria italica
Source: BMC Plant Biol. 2022 Nov 28;22:547. doi: 10.1186/s12870-022-03921-1 (PMC9703814; doi:10.1186/s12870-022-03921-1)
Supplement: Supplementary file 9 — Additional file 9. Expression patterns of some duplicated SinsLTP genes after drought (a, b), salt (c, d) and cold stress treatment (e, f). [file 12870_2022_3921_MOESM9_ESM.docx]

e

d

b

a


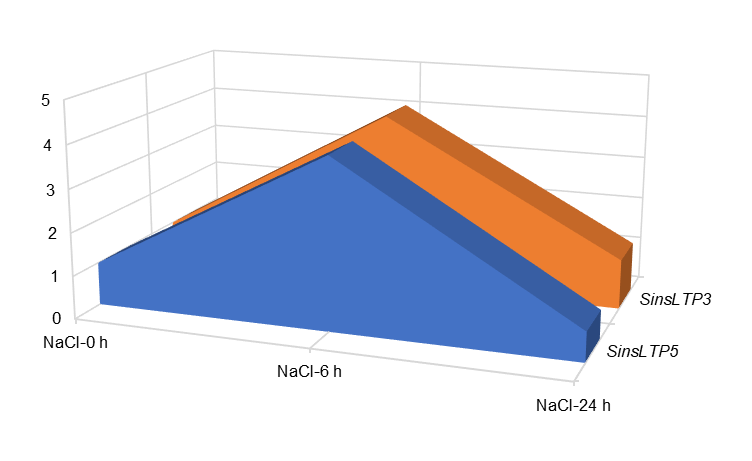

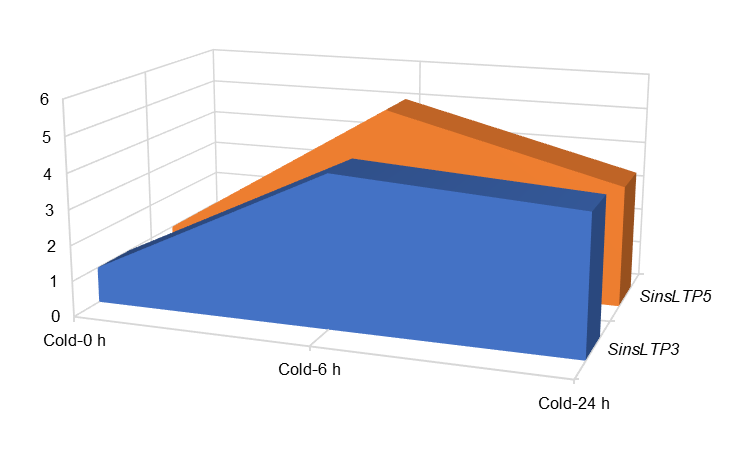

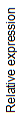


c


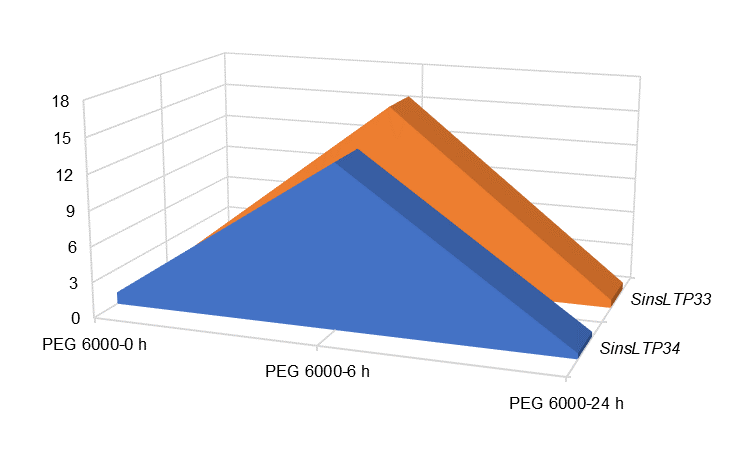

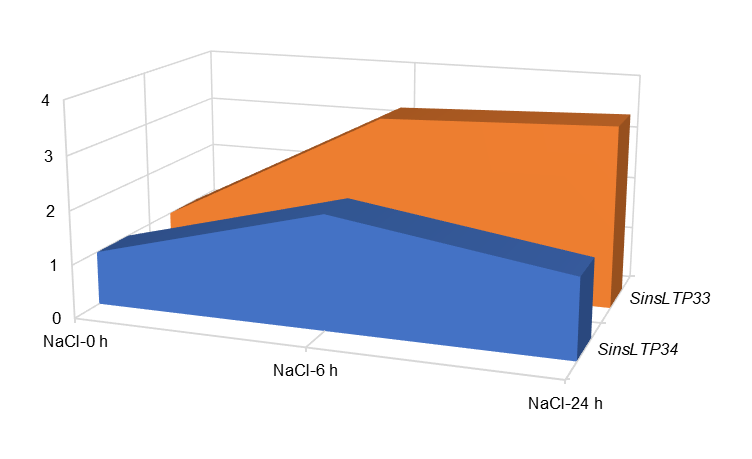

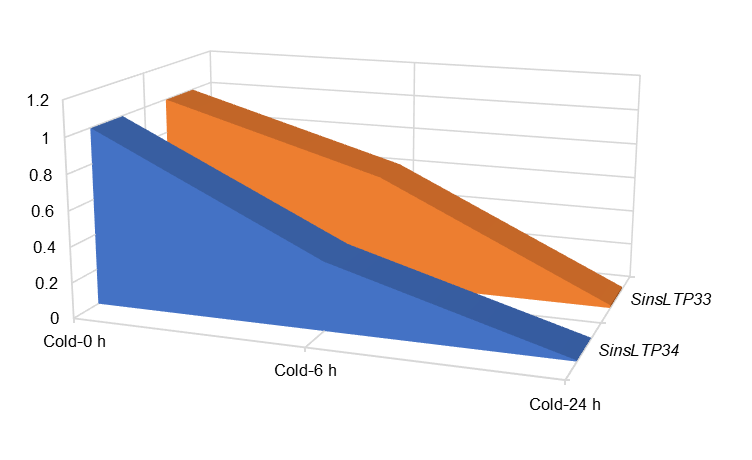

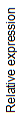


f


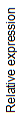

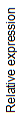

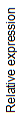

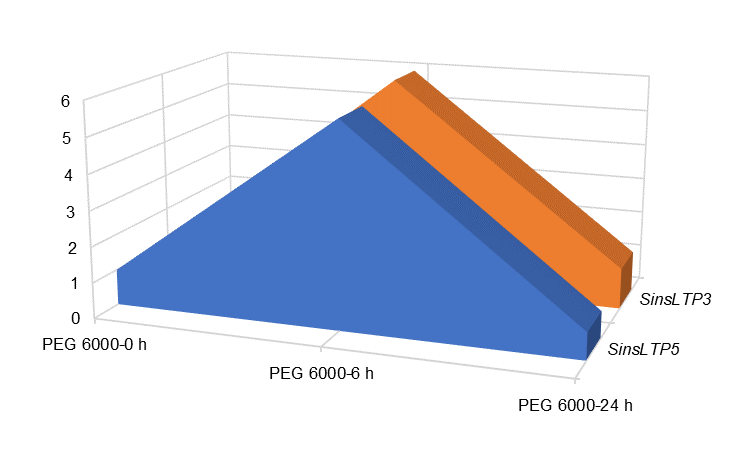

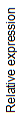


**Additional file 9:** Expression patterns of some duplicated *SinsLTP* genes after drought (**a, b**), salt (**c, d**) and cold stress treatment (**e, f**).
