# Supplementary material for: Genome-wide identification, molecular evolution and expression analysis of the non-specific lipid transfer protein (nsLTP) family in Setaria italica
Source: BMC Plant Biol. 2022 Nov 28;22:547. doi: 10.1186/s12870-022-03921-1 (PMC9703814; doi:10.1186/s12870-022-03921-1)
Supplement: Supplementary file 10 — Additional file 10. PCR primers used for qRT-PCR in this study. [file 12870_2022_3921_MOESM10_ESM.docx]

**Additional file 10:** PCR primers used for qRT-PCR in this study

| Gene name | Forward primer (5'-3') | Reverse primer (5'-3') |
| --- | --- | --- |
| *Actin* | CACCACCTGAGAGGAAATACAG | GACTCATCATACTCACCCTTCG |
| *SinsLTP2* | ACGAGCAGTTCATGTCGTG | TGTTGTACACGCCCATCC |
| *SinsLTP3* | TGTGCCGGTACAAGAACG | GCATCGCAAGACCACACT |
| *SinsLTP5* | AAGGCACAGGCAATGGG | GCTCGACAGGTTGCAGAT |
| *SinsLTP9* | CAGCAAGATGAGGGAGCAG | ATGGCCTTCTTGCCGTT |
| *SinsLTP15* | GGAGGTCGCCATTACCATC | GTGTAGTCTCCGCACTTGTAG |
| *SinsLTP21* | GCTCCGGCACGTACATC | TTGCAGTTGACGCTGGT |
| *SinsLTP33* | GGCACCATCAAGAAGCTCAA | TTAGCTGACCTTGTTGCAGTC |
| *SinsLTP34* | ATGGCTCCGATGAGGAAGAT | ACCTGACCGCAGGTGAT |
| *SinsLTP40* | GGTTGCCTCTGCGTGTA | TAGGCGACCTGGCAGTA |
| *SinsLTP42* | AGTTTCAGGCCAACCATCTC | AAGACGGCTTCTGCTTGG |
